# Identification and Characterization of Mediators of Fluconazole Tolerance in *Candida albicans*

**DOI:** 10.3389/fmicb.2020.591140

**Published:** 2020-11-11

**Authors:** Eric Delarze, Ludivine Brandt, Emilie Trachsel, Marion Patxot, Claire Pralong, Fabio Maranzano, Murielle Chauvel, Mélanie Legrand, Sadri Znaidi, Marie-Elisabeth Bougnoux, Christophe d’Enfert, Dominique Sanglard

**Affiliations:** ^1^Department of Laboratory, Institute of Microbiology, Lausanne University Hospital, Lausanne, Switzerland; ^2^Department of Computational Biology, University of Lausanne, Lausanne, Switzerland; ^3^Unité Biologie et Pathogénicité Fongiques, Département de Mycologie, Institut Pasteur, USC 2019 INRA, Paris, France; ^4^Unité de Parasitologie-Mycologie, Service de Microbiologie Clinique, Hôpital Necker-Enfants-Malades, Assistance Publique des Hôpitaux de Paris (APHP), Paris, France; ^5^Université de Paris, Paris, France

**Keywords:** *Candida*, fluconazole, drug resistance, drug tolerance, calcineurin

## Abstract

*Candida albicans* is an important human pathogen and a major concern in intensive care units around the world. *C. albicans* infections are associated with a high mortality despite the use of antifungal treatments. One of the causes of therapeutic failures is the acquisition of antifungal resistance by mutations in the *C. albicans* genome. Fluconazole (FLC) is one of the most widely used antifungal and mechanisms of FLC resistance occurring by mutations have been extensively investigated. However, some clinical isolates are known to be able to survive at high FLC concentrations without acquiring resistance mutations, a phenotype known as tolerance. Mechanisms behind FLC tolerance are not well studied, mainly due to the lack of a proper way to identify and quantify tolerance in clinical isolates. We proposed here culture conditions to investigate FLC tolerance as well as an easy and efficient method to identity and quantify tolerance to FLC. The screening of *C. albicans* strain collections revealed that FLC tolerance is pH- and strain-dependent, suggesting the involvement of multiple mechanisms. Here, we addressed the identification of FLC tolerance mediators in *C. albicans* by an overexpression strategy focusing on 572 *C. albicans* genes. This strategy led to the identification of two transcription factors, *CRZ1* and *GZF3*. *CRZ1* is a C2H2-type transcription factor that is part of the calcineurin-dependent pathway in *C. albicans*, while *GZF3* is a GATA-type transcription factor of unknown function in *C. albicans*. Overexpression of each gene resulted in an increase of FLC tolerance, however, only the deletion of *CRZ1* in clinical FLC-tolerant strains consistently decreased their FLC tolerance. Transcription profiling of clinical isolates with variable levels of FLC tolerance confirmed a calcineurin-dependent signature in these isolates when exposed to FLC.

## Introduction

*Candida* species are major fungal pathogens which can cause life-threatening systemic infection known as invasive candidiasis. *Candida* infection are linked to a high mortality rate despite treatments with antifungals. Therapeutic failures might be partially explained by acquisition of resistance upon treatment (Pfaller and Diekema, 2010). Azoles, and particularly fluconazole (FLC), are still widely used in the management of fungal infection including *C. albicans* infections (Berman and Krysan, 2020). Resistance mechanisms to FLC have been extensively studied (Morschhäuser, 2016). However, despite an extensive knowledge of these mechanisms, the acquisition of resistance does not explain all therapeutic failures. Indeed, it has been observed that some isolates identified as susceptible to FLC *in vitro* cannot be treated efficiently in the patient. It is known that some clinical isolates of *C. albicans* tolerate the presence of FLC more than others. These are characterized by an increased growth, known as residual growth, at drug concentrations above their minimum inhibitory concentration (MIC). This residual growth, which is a characteristic of drug tolerance, is in general not coupled to an increased MIC (unlike resistance) and is thought to favor the acquisition of resistance mechanisms due to extended survival of some *C. albicans* isolates upon treatment. Tolerant isolates may also to be a source of reinfection upon arrest of antifungal treatment (Delarze and Sanglard, 2015).

A few mechanisms have been linked to FLC tolerance in *C. albicans* in the past, even if a clear definition of tolerance was not yet available. It was demonstrated that the immunosuppressive drug cyclosporine could convert the fungistatic drug FLC into a strong fungicidal agent *in vitro*, thus reflecting a loss of tolerance (Sanglard et al., 2003). Cyclosporine A, associated with cyclophilin A (*CYP*), targets calcineurin, which is a protein phosphatase activated by a calcium-dependent pathway (Sanglard, 2003). Exposure of the calcineurin subunit A (*CNA*/*CMP1*) deletion mutant to FLC results in a strong loss of viability reflecting a loss of tolerance for the drug. In contrast, the deletion of the *CYP* gene was viable in presence of FLC and cyclosporine A. Calcineurin regulates drug tolerance only and not resistance, since the presence or absence of its activity does not affect drug susceptibility in drug susceptibility assays (Sanglard et al., 2003). The calcineurin pathway leads to the activation of a transcription factor (*CRZ1*), which itself was reported to modify FLC tolerance (Onyewu et al., 2004; Karababa et al., 2006). Calcineurin was also shown to be a client protein of the chaperone heat shock protein 90 (Hsp90) (Cowen, 2008). Inhibition of Hsp90 results in a loss of calcineurin activity and, therefore, in a loss of FLC tolerance such as observed with the *CNA* deletion mutant (Cowen, 2013). In another study (Luna-Tapia et al., 2015), it was shown that the membrane trafficking through the late endosome/pre-vacuolar compartment (PVC) had an impact on *C. albicans* FLC tolerance. Deletion of *VPS21*, an endosomal Rab family GTPase involved in PVC trafficking, resulted in enhanced growth in the presence of FLC. This enhanced growth appeared as trailing growth in standard susceptibility assays, thus pointing to a potential role of Vps21 in azole tolerance. In addition, the *VPS21* mutant contained abnormally high levels of intracellular Ca^2+^, thus potentially increasing the basal level of calcineurin expression and/or activation (Luna-Tapia et al., 2015). It has also been observed that Rta3, a putative lipid translocase, is co-upregulated with multidrug transporters by the transcription factor *TAC1* in resistant *C. albicans* strains. While deletion of *RTA3* increases the susceptibility to FLC, overexpression of *RTA3* results in an increase of tolerance characterized by a trailing growth in the susceptible *C. albicans* strain SC5314 (Whaley et al., 2016). Recently, it was reported that *FEN1* and *FEN12*, two *C. albicans* genes involved in sphingolipid biosynthesis, may be involved in FLC tolerance. Indeed, deletion of *FEN1* or *FEN12* resulted in increased tolerance in the presence of FLC. The increased survival of the mutants was linked to an increased intracellular FLC concentration and a decreased level in the plasma membrane of the toxic sterol 14-methylergosta-8,24(28)-dien-3,6-diol responsible for the toxic effect of FLC in *C. albicans*. These modifications in toxic sterol concentrations were reflected by a high level of sphingolipids which improved cell membrane strength (Gao et al., 2018). Another recently published study highlighted a tolerance mechanism involving the pH response via the Rim pathway. This pathway includes Rim9, Rim21/Dfg16, Rim8, Rim20, and Rim13. Under neutral-alkaline pH, this cascade leads to the cleavage of the C-terminal inhibitory domain of the transcription factor Rim101 leading to its activation. The culture medium’s pH was already known to impact the level of tolerance *in vitro* (Marr et al., 1999) and it has been observed that tolerance decreases under acidic conditions, while it is not affected in neutral conditions (pH 6–7). A phenotypic and transcriptomic analysis of the Rim pathway showed that all Rim proteins were important for FLC tolerance since their respective deletion mutants exhibited increased FLC susceptibility (Garnaud et al., 2018). The transcriptomic comparison between a *RIM101* deletion mutant and a wild type strain at alkaline and acidic pH led to the identification of two Rim101 downstream targets, including *HSP90* and *IPT1. HSP90* is already known to be involved in antifungal tolerance on the opposite to *IPT1*, which encodes an enzyme involved in the biosynthesis of the most abundant sphingolipid in the plasma membrane [mannose-(inositol-P)2-ceramide] (Garnaud et al., 2018). Interestingly, Ipt1 is part of the sphingolipid’s biosynthesis pathway and is situated downstream of Fen1 and Fen12, both of which were reported as FLC tolerance mediators (see above). These data thus reinforce the idea that sphingolipids homeostasis is of relevance for FLC tolerance in *C. albicans*. In addition to these genotypic/phenotypic relationships, recent studies highlighted that FLC tolerance can develop in clinical strains from distinct subpopulations (Rosenberg et al., 2018).

Even if the above-listed mechanisms may lead to azole tolerance, the exact molecular basis of tolerance is still not completely understood. Moreover, most of these mechanisms have not been proven to mediate tolerance in clinical isolates. Tolerance may allow a better survival of *C. albicans* at high drug concentrations. The strong selective pressure induced by administered drugs and persistence resulting from residual growth may favor the acquisition of resistance mechanisms and may ultimately lead to treatment failure. Unraveling genetic and epigenetic mechanisms underlying tolerance and the discovery of tolerance mediators are essential steps for the development of potential inhibitors that, in combination with FLC, might offer new strategies to fight against *Candida* infections.

In this study, we first adapted a standardized microdilution method to measure tolerance in a subset of *C. albicans* clinical isolates. We next addressed the identification of mediators of FLC tolerance using a gene overexpression strategy in a laboratory *C. albicans* strain. This strategy has been already effective in the identification of genes associated with biofilm formation and gut colonization (Znaidi et al., 2018). Starting with a limited pool of *C. albicans* genes, we were able to identify at least two transcription factors (*CRZ1*, *GZF3*) as mediators of FLC tolerance. One of them, *CRZ1*, a transcription factor under the control of the calcium-dependent calcineurin pathway, was shown as critical in the maintenance of FLC tolerance of several clinical strains. Consistent with this finding, we showed by transcriptional analysis of clinical strains that the calcium-dependent calcineurin pathway was stimulated by FLC exposure.

## Materials and Methods

### Strains Used in This Study

The wild type (WT) strain SC5314 and the *C. albicans* clinical isolates used in this study were selected for their known susceptibility to FLC and belong to D. Sanglard’s lab collection (Supplementary File S1). SSI strains were kindly provided by M. C. Arendrup (Staten Serum Institute, Copenhagen, Denmark) and M. E. Bougnoux (Institut Pasteur, Paris, France) provided the CEC strain collection (Supplementary File S1). The collection of 572 Tet-inducible overexpression barcoded and detectable *C. albicans* strains used in this study was kindly provided by C. d’Enfert (Supplementary File S2). Each strain of the collection overexpresses a specific gene (or gene of interest, GOI) encoding transcription factors, protein kinases, protein phosphatases, proteins related to DNA replication, recombination and repair, predicted cell surface proteins, or others, under the control of tetracycline. The collection was constructed as described by Chauvel et al. (2012). To test optimal overexpression culture condition, the strain CEC3083 was used which overexpresses the *Gaussia princeps* luciferase (*gLUC*). This strain was built in parallel to the OE collection and also provided by C. d’Enfert (Supplementary Table S1) (Chauvel et al., 2012). The clinical *C. albicans* isolates used for the overexpression and deletion of the candidate regulators of tolerance were part of D. Sanglard’s laboratory collection (Supplementary Table S1). *CRZ1* and *GZF3* deletion mutants as well as the double *crz1*Δ/Δ, *gzf3*Δ/Δ mutants originating from the Homann’s transcription factor deletion library are listed in Supplementary Table S1 (Homann et al., 2009). *Escherichia coli* DH5α was used as a host for plasmid construction and propagation.

### Media and Growth Conditions

All fungal strains were stored in 20% glycerol at −80°C and routinely grown at 30°C under constant shaking (220 rpm) in complete Yeast Extract Peptone Dextrose (YEPD) medium (1% Bacto peptone, Difco Laboratories, Basel, Switzerland), 0.5% Yeast extract (Difco) and 2% glucose (Fluka, Buchs, Switzerland). When grown on solid YEPD plates (YEPDA), 2% agar (Difco) was added and plates were incubated at 35°C. Bacteria were grown in Luria-Bertani (LB) broth or LB 2% agar plates, supplemented with 100 μg/ml ampicillin (AppliChem, Darmstadt, Germany) or 34 μg/ml chloramphenicol (Fluka) when required and incubated at 37°C.

For FLC susceptibility assays, strains were grown according to the EUCAST or CLSI recommendations (Clinical and Laboratory Standards Institute [CLSI], 2008; European Committee on Antimicrobial Susceptibility Testing, 2017) in RPMI1640 medium with phenol-red (Sigma-Aldrich, St. Louis, United States), complemented with 0.2 or 2% glucose (Fluka) and buffered with 0.33 M morpholinepropanesulfonic acid (MOPS). The pH was adjusted to pH 7.5, pH 7, pH 6, and pH 4.5, respectively, using NaOH or HCl. Stock solutions were prepared as 2x concentrated RPMI as recommended by EUCAST (European Committee on Antimicrobial Susceptibility Testing, 2017). RPMI was supplemented or not with FLC (Toronto Research Chemicals, North York, Canada), doxycycline (Dox) (Sigma-Aldrich) and iron (FeCl_3_) to compensate iron chelation by Dox when necessary (Fiori and Van Dijck, 2012). For luciferase assays, water-soluble coelenterazine (CTZ, NanoLight Technology, Prolume Ltd. Pinetop, United States) was added prior to measurement.

### Primers and Plasmids

All primers used in this study are listed in Supplementary File S3. Plasmids used in this study are listed in Supplementary File S4. The insertion of each plasmid and gene deletion were verified by PCR as stated in Supplementary File S3.

### FLC Susceptibility and Tolerance Assays

All FLC susceptibility assays were performed according to the Reference Method for Broth Dilution Antifungal Susceptibility Testing of Yeasts (M27-A3) (Clinical and Laboratory Standards Institute [CLSI], 2008) and the method for the determination of broth dilution MICs of antifungal agents for yeasts (E.DEF 7.3.1) (European Committee on Antimicrobial Susceptibility Testing, 2017) with slight modifications when necessary. Susceptibility assays were performed in flat-bottom Costar^®^ 96-well plates (Corning Inc, Corning, United States) and the fungal growth was measured spectrophotometrically for both methods. The minimal inhibitory concentration (MIC) was defined as the first concentration of drug inhibiting at least 50% of fungal growth as compared to a drug free control. Strains with a MIC below 4 μg/ml FLC were defined as susceptible as described by EUCAST (European Committee on Antimicrobial Susceptibility Testing, 2017). Susceptibility assays were performed in RPMI at pH 7.5, pH 7, pH 6 or pH 4. Tolerance assays were performed in the same conditions but using one fixed only at FLC concentration as specified in the corresponding experiment.

Briefly, individual colonies of each tested strain were grown overnight in YEPD medium at 30°C under constant shaking (220 rpm). Cultures were centrifuged (5 min, 4600 rpm, 4°C) and washed twice with PBS (137 mM NaCl, 10 mM phosphate, 2.7 mM KCl, and pH 7.4). Cell concentration was estimated by spectrophotometry (OD_540 nm_) and cells resuspended at 5 × 10^5^ cells/ml in distilled water. Then, 100 μl of this suspension were transferred into the wells of flat-bottom 96-well plates containing 100 μl of 2x RPMI (0.2 or 2% glucose for CLSI and EUCAST condition, respectively) complemented with 256–0 μg/ml FLC in two-fold dilutions or fixed FLC concentrations, to obtain a final cell concentration of 2.5 × 10^5^ cells/ml and FLC concentrations ranging from 128 to 0 μg/ml (8 μg/ml for tolerance assays) per well. Plates were incubated at 35°C without shaking for 24 h. Cell growth in each well was measured by spectrophotometry (OD_570 nm_) using a microplate reader (Multiskan Ascent, Thermo Scientific) and compared to the drug-free control after correction with a blank control. From these susceptibility curves, both the MIC and the tolerance index (TI), corresponding to the relative growth (% GC) at 8 μg/ml FLC divided by a factor 100 (TI = % GC/100) were extracted for strain comparisons. The TI is used to determine the level of tolerance of the tested strains.

To assess the tolerance level of every single strain of the OE collection and to remain in the same conditions compared to the enrichment assay, tolerance assays of the collection were performed at a final concentration of 32 μg/ml FLC instead of 8 μg/ml.

### Construction of Deletion Mutants and Revertant Strains

The clinical isolates DSY2110, DSY4454, SSI 4622, and DSY4588 as well as the “WT,” the *crz1*Δ/Δ and the *gzf3*Δ/Δ strains from the Homann’s collection (Homann et al., 2009) were used to delete: (i) *URA3*, in order to transform the strains with the OE system (Chauvel et al., 2012) and (ii) either *CRZ1*, *GZF3* or both to assess their role in FLC tolerance. All gene deletions were performed by insertion of the PCR amplified 5′- and 3′- untranslated regions (UTR, 500 bp) into the *SAT1*-flipper plasmid pSFS2A (Reuss et al., 2004). Details for the resulting plasmids could be found in Supplementary File S4. *URA3* was deleted using the plasmid pSFSU1 (Supplementary File S4). Due to technical issues with some clones, the second allele was deleted by plating the heterozygous mutants on 5-fluoro-orotic acid (5-FOA) to force deletion of the second allele as already described (Wellington and Rustchenko, 2005). For deletion of *CRZ1* and *GZF3*, the plasmids pED19-2, pED20-7 and pET2-1 were used, respectively (Supplementary File S4). The plasmid pET2-1 was only used to delete the second *GZF3* allele in DSY5217, resulting in strain DSY5252.

All plasmids were transformed into their target strains after digestion by *Kpn*I and *Sac*I to isolate the *SAT1* flipper-cassette. The transformants were plated and selected on YEPD plates containing 200 μg/ml nourseothricin. The cassette was flipped-out as described earlier (Reuss et al., 2004). To verify gene deletions in selected isolates and the presence of the wild type alleles, gene-specific primers were used in PCR (Supplementary File S3).

To construct revertant strains in which *CRZ1* and *GZF3* were reintroduced in the background of deletion mutants, the wild type alleles from SC5314 and corresponding 5′- and 3′-UTR (±500 bp) were reinserted by replacing the 5′-UTR of the deletion plasmid by the *CRZ1* and *GZF3* amplified genes, resulting in plasmids pLB1 and pET3, respectively (Supplementary File S4). The wild type alleles were then reintroduced in addition to the *SAT1*-flipper cassette into the homozygous mutants by *Kpn*I and *Sac*I digestion of the plasmids.

### Determination of Optimal Dox Concentration for OE

To optimize the culture conditions for overexpression with the OE collection, the ability of Dox to induce OE was evaluated using the *gLUC*-OE strain CEC3083 (Supplementary Table S1). Briefly, individual colonies were grown o/n in liquid YEPD medium at 30°C under constant shaking. The culture was then centrifuged (5 min, 4600 rpm, 4°C) and the cell pellet washed twice with phosphate buffered saline (PBS). Cells were then resuspended at 7.5 × 10^6^ cells/ml in RPMI medium at pHs 7.5, 7, 6, and 4.5. Each inoculum was split in five subcultures and complemented with 100, 50, 10 and 2 and 0 μg/ml Dox, respectively. Cultures were then incubated for 24 h at 30°C under constant shaking. The next day, 50 μl of each culture were transferred into the wells of a flat-bottom, half-area, black microtiter plate. The plates were placed in a luminometer (FLUOstar Omega, BMG Labtech, Ortenberg, Germany) and 50 μl of 2.5 μM water soluble CTZ injected prior to the measurement of luciferase activity of each well.

### Yeast Transformation

Yeasts were transformed by a lithium-acetate procedure as previously described (Sanglard et al., 1996).

### Pool Enrichment in Tolerant/Resistant Strains

In order to enrich the pooled OE collection in FLC tolerant/resistant isolates, strains were grown in RPMI 2% glucose at pH 6 in the presence of 1 mM FeCl_3_ and with or without 100 μg/ml Dox and 32 μg/ml FLC. To be as close as possible to our drug susceptibility assay conditions (EUCAST recommendations), cells were cultured in 96-well plates with each well containing 200 μl of cell suspension in RPMI. Each condition represents 20 ml of culture.

Briefly, a pool of the 572-strain collection was incubated overnight in YEPD at 35°C under constant shaking. The next day, cells were centrifuged (5 min, 4600 rpm, 4°C) and washed twice with PBS. The cell pellet was resuspended at 10^7^ cells/ml in RPMI pH 6 and this inoculum was split into four 20 ml subcultures. The remainders of the overnight culture were centrifuged and the pellet frozen (−20°C) prior to DNA extraction at the end of the enrichment assay to estimate the initial population of our pool (Day 0). The four subcultures were respectively complemented with: (i) 32 μg/ml FLC, (ii) 100 μg/ml Dox, (iii) 32 μg/ml FLC and 100 μg/ml Dox. The last subculture was the growth control in which nor FLC or Dox were added. Each subculture was also complemented with 1 mM FeCl_3_ to avoid the known synergistic FLC-Dox effect (Fiori and Van Dijck, 2012). Two hundred microliter of these subcultures were then transferred in each well of 96-well plates, leaving two wells as contamination control. The plates were then incubated 24 h at 35°C. Every day for the next 5 days, cells from the overnight culture in 96-well plates were recovered using a multi-channel pipette and recovered into a 50 ml tube. The cultures were then processed as at Day 0. First, the cells were centrifuged (5 min, 4600 rpm, 4°C) and washed twice with PBS and resuspended at 10^7^ cells/ml in 20 ml fresh RPMI in four new 50 ml tubes prior to addition of FLC, Dox and FeCl_3_. The rest of the overnight cultures were centrifuged once more and the pellet frozen (−20°C) until DNA extraction.

### Genomic DNA Extraction

Genomic DNA was prepared by spheroplasting from frozen cell pellets according to Calabrese et al. (2000).

### Amplification of the Barcodes

In order to estimate the strain presence in the different populations, the BC of each cell was amplified by PCR to be sequenced following the *16S Metagenomic Sequencing Library Preparation protocol* (Illumina, Inc., San Diego, United States) recommendations to add so-called Illumina overhang sequences to the amplicons (Supplementary File S3). However, to avoid mistakes during the sequencing due to the similar flanking regions of the BC amplicons, spacer bases (0, 4, 8, and 12 N) were added to the forward primers to shift the signal during sequencing (Supplementary File S3). Briefly, approximatively 12.5 ng of DNA extraction was used for BC amplification using the Phusion High-Fidelity DNA Polymerase (New England Biolabs, Ipswich, United States) following manufacturer recommendations. The only modification to the preparation of the samples was to use four forward primers instead of one (Supplementary File S3), each diluted to a 1:4 ratio. The PCRs were performed in a peqSTAR 2x thermal cycler (Peqlab-VWR, Radnor, United States) with the following program: 3 min at 95°C followed by 30 cycles at 95°C, 57.9°C and 72°C for 30 s each. A final elongation was performed for 5 min at 72°C. To increase the quality of our samples, the whole PCR products were loaded on a 1% agarose gel for electrophoresis and bands of interest purified using the NucleoSpin^®^ Gel and PCR Clean-up kit (Macherey-Nagel, Düren, Germany) following manufacturer recommendations.

### Library Preparation and Sequencing

Library construction was performed at the Lausanne Genome Technologies Facility (LGTF, University of Lausanne – Centre for Integrative Genomics, Lausanne, Switzerland). Libraries were prepared adapting the *16S Metagenomic Sequencing Library Preparation protocol* (Illumina) according to manufacturer recommendations. MiSEQ Illumina 300 bp single-end sequencing was performed on the libraries by the LGTF.

### Barcode Quantification

The barcode quantification of the populations from the pool enrichment assay was performed using a script developed by Marion Patxot^1^. Each sequenced library was outputted as a FASTQ file. To process the sequencing data, a bioinformatics pipeline was set up using Perl programming language (v. 5.24.0) and bash Shell (v. 3.2.57 for Mac OS X 10.11.16). Briefly, the pipeline counts the occurrence of known barcode sequences in a FASTQ file by running a MegaBLAST. There are two inputs to the pipeline: the FASTQ file and a text file containing the name and sequence of each barcode. Both files are first converted into FASTA format. Each barcode sequence is then aligned to each read in the FASTQ file. The alignment allowed 8 mismatches. The pipeline outputs the occurrence of each barcode as a text file. The sequence data was processed using the developed pipeline. Counts for each time point and condition were merged into a single text file with bash Shell. The proportion of each strain in the population was subsequently calculated and plotted using Open Source R Software (v. 3.2.4)^2^. In a second step, counts were analyzed using the WT statistical guideline for DMS studies described by Matuszewski et al. (2016). This approach calculates a selection of coefficient estimators using the log ratios of the number of counts for strains over the number of counts for the reference.

### RNA Extraction

RNA was extracted according to Amorim-Vaz et al. (2015). Briefly, individual colonies of each tested strain were grown overnight in YEPD medium at 30°C under constant shaking (220 rpm). Cultures were centrifuged (5 min, 4600 rpm, 4°C) and washed twice with PBS (137 mM NaCl, 10 mM phosphate, 2.7 mM KCl, and pH 7.4). Cell concentration was estimated by spectrophotometry (OD_540 nm_) and cells were resuspended at 5 × 10^5^ cells/ml in distilled water. Hundred μl of this suspension were next transferred into the wells of flat-bottom 96-well plates containing 100 μl of 2x RPMI (2% glucose) complemented or not with 16 μg/ml FLC to obtain a final cell concentration of 2.5 × 10^5^ cells/ml and 8 μg/ml FLC per well. Each tested condition corresponded to a whole 96-well plate and represented approximatively 18.8 ml of culture in total. Plates were incubated at 35°C without shaking for 24 h. All nucleic acids were extracted by mechanical disruption using glass beads and phenol-chloroform separation as described previously (Amorim-Vaz et al., 2015). The nucleic acids were stored at −80°C prior to DNase treatment. Genomic DNA was removed by DNase treatment using the DNA-free DNA Removal Kit (Thermo-Fisher Scientific) following manufacturer’s recommendation and samples were stored at −80°C.

### RNA Sequencing and RNAseq Data Mining

Library preparation and sequencing were carried out by the LGTF and the sequencing was performed on an Illumina HiSeq 2500 system (Illumina), using the TruSeq Stranded RNA, 125 bp single read protocol. The analysis was performed with three biological replicates for each condition. RNAseq data were processed using CLC Genomic Workbench Version 10.1.1 (Qiagen, Hilden, Germany). Reads were aligned to the *C. albicans* SC5314 genome and read counts were normalized using the quantile approach method. All conditions were compared with each other and filtered according to a specific statistical cut-off as explained in the Results section. Raw data can be accessible under the Bioproject numbers PRJNA638239 and PRJNA638870.

### Software and Statistical Analysis

Graphics and statistical analysis were performed in GraphPad Prism 7.03 (GraphPad Software, Inc., La Jolla, United States). The online software Morpheus^3^ was used to create heatmaps for the tolerance profile screening.

## Results

### Optimizing Culture Condition for FLC Tolerance Identification

Several techniques are available to establish the antifungal susceptibility profiles of defined *Candida* isolates. Most of them are based on drug diffusion assays in either a solid agar medium or in liquid conditions, with each protocol having its specific endpoint readout (Pappas et al., 2018). Despite having the same purpose (i.e., identifying susceptible and resistant isolates), each method has its advantages and flaws. Indeed, depending on the *Candida* species and the tested drug, a given protocol might be more optimal than another. A method for the identification and quantification of tolerance to FLC on agar medium has already been proposed (Gerstein et al., 2016). We here focused on the identification of tolerance using standardized susceptibility assays in microtiter plates (proposed by the EUCAST and the CLSI) as it could be implemented and reproduced in most laboratories.

**FIGURE 1:**
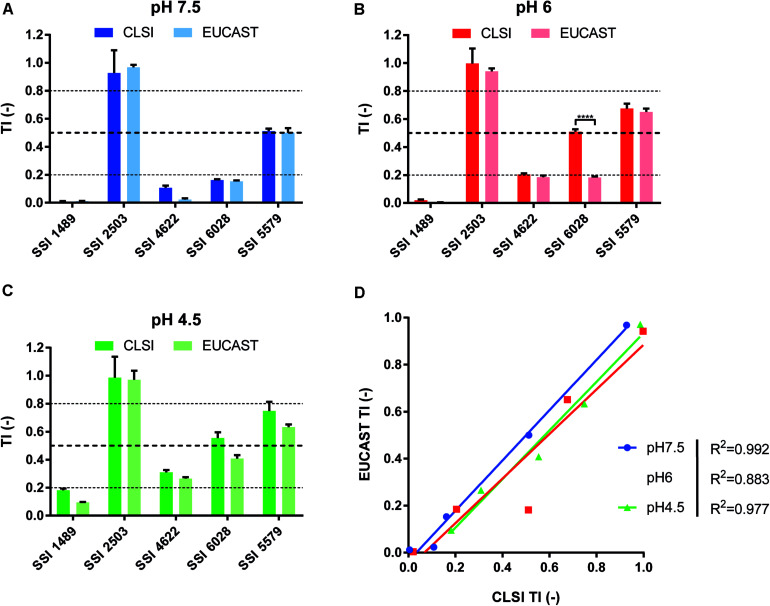
Comparison of CLSI and EUCAST protocols for the identification of tolerant isolates. Comparison was performed at pHs 7.5, 6.0, and 4.5 (**A–C**, respectively) to assess the pH effect upon tolerance as stated by Marr et al. (1999). **(D)** Represents the correlation between both approaches with the respective *R*^2^ corresponding to each tested pH (Two-tailed Pearson correlation. confidence interval = 95%). Each bar corresponds to the mean of biological duplicates. Statistical significance was calculated using Sidak’s multiple comparison tests (two-way ANOVA, 95% confidence interval). *P*-values: <0.0001 (****).

We first performed drug susceptibility assays of several clinical isolates known to exhibit FLC resistance and/or different residual growth pattern (or “trailing”) in FLC susceptibility EUCAST and CLSI tests with slight modifications. Trailing growth will be now referred as a phenomenon related to tolerance. We also performed FLC susceptibility assays at pH values of 7.5, 6, and 4.5, since it has been reported that the pH of the culture medium might influence the level of tolerance (Marr et al., 1999). The results showed that both methods were comparable in terms of strain categorization. The tested strains clearly exhibited increased FLC tolerance when the growth medium turned acidic (Supplementary Figure S1). The tested clinical isolates SSI 1489, SSI 4622, and SSI 6028 could be categorized as susceptible in all the tested conditions, as represented by their MICs below the clinical breakpoint (CBP) of 4 μg/ml for FLC in *C. albicans*, with the exception of isolate SSI 2503, which was known as FLC-resistant (Supplementary File S1). Isolate SSI 5579 was more ambiguous since its relative growth lied around the 50% relative growth threshold at inhibitory FLC drug concentrations. Growth reduction could still be observed at low FLC concentrations (0–0.125 μg/ml FLC) and thus indicated that this strain might not be considered as resistant, but rather as highly tolerant to FLC. To summarize, each strain clearly exhibited a different level of tolerance above the MIC, ranging from low values of relative growth (1–2% as compared to drug-free control) to around 50% depending on the strain and the tested condition (Supplementary Figure S2). We reflected these tolerance levels by the use of a so-called tolerance index [TI = relative growth (%)/100] based on growth measurement of the strains at a fixed supra-MIC FLC concentration of 8 μg/ml (Figure 1).

To help distinguish between wild type, tolerant and resistant isolates, we proposed to set TI thresholds. We first considered that strains without decreased growth above the CBP as resistant. On the other hand, we considered isolates as wild type when residual growth above their MIC would not exceed 20% or relative growth (TI < 0.2). Taking the results of Supplementary Figure S1 as guides, we proposed to assign strains as tolerant when their relative growth ranged between 20 and 80% (TI ≥ 0.2 and ≤ 0.8). Even though these TI thresholds are not based on the occurrence of molecular markers of tolerance, which are still to be discovered, they still allow a discrimination between phenotypes that will be useful in this study by using a single FLC concentration (8 μg/ml) to facilitate strain comparisons and large-scale screenings.

### Application of the Tolerance Assay for the Identification of Tolerant Strains

**FIGURE 2:**
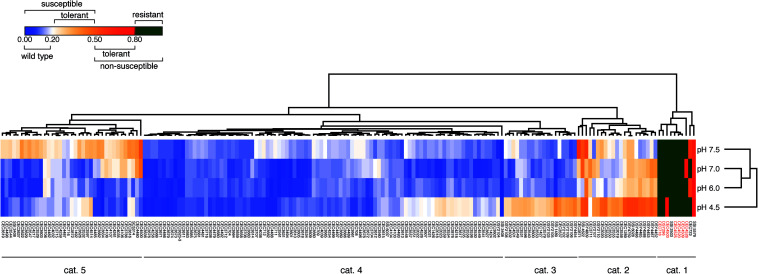
FLC tolerance screen of 181 *C. albicans* strains. The heatmap represents the mean TI values of at least 2 biological replicates. The “susceptible” and “non-susceptible” categories of the legend are based on the EUCAST definitions. Detailed results can be found in Supplementary File S1. Heatmap was generated with Morpheus (https://software.broadinstitute.org/morpheus/) with Euclidian distance metrics and average linkage method including hierarchical clustering on TI values and pH conditions. Red labeled isolate names correspond to isolates with known azole resistance mechanisms (Supplementary File S1).

To confirm the efficiency of the TI to identify tolerant strains, we next performed a large screening of *C. albicans* clinical isolates from different origins (*n* = 181) including isolates with known acquired azole resistance mechanisms. Data summarized as a heat map in Figure 2 show that 5 major categories of phenotypes could be distinguished according to the obtained TIs. A first category (Figure 2, category 1) contained mainly azole-resistant strains above the 0.8 threshold TI value at all tested pH values. Since the majority of these strains contain known azole resistance mechanisms (Supplementary File S1), this validates the selection of the TI threshold value at 0.8 for azole-resistant strains. A second category (Figure 2, category 2) contained isolates exhibiting tolerance at specific pHs. A third category (Figure 2, category 3) contained isolates exhibiting tolerance at low pH (pH 4.5). A fourth category (Figure 2, category 4) contained azole-susceptible isolates with wild type characteristics (TI threshold ≤ 0.2). The last and fifth category contained isolates exhibiting tolerance at high pHs only (pH 7.0–7.5). No correlations were identified between clade assignments of tested strains and TI profiles. The diversity in FLC tolerance patterns suggests that multiple tolerance mechanisms could be involved.

### Gene Overexpression Strategy for the Identification of FLC Tolerance Mediators

In order to identify genes involved in FLC tolerance, we chose a gene overexpression strategy. To achieve this purpose, a 572-strains collection reported in Chauvel et al. (2012) and Znaidi et al. (2018) was used, with each strain overexpressing a specific gene on a Dox-dependent manner. Briefly, the OE system consists of two distinct plasmids. First, a vector (pNIMX) expressing the Tetracycline (TET)-binding transactivator (TA) under the control of the *TDH3* promoter (P*_TDH3_*) was introduced in the parental strain CEC161, which is derived from BWP17, yielding strain CEC2097 (Chauvel et al., 2012). Next, the CIp10-P_TET_ plasmid collection (572 plasmids), with each plasmid expressing a gene of interest (GOI, listed in Supplementary File S2) under the control of a TET-inducible promoter (P_TET_) was introduced in CEC2097. These second plasmids also bear specifics 20 bp tags (or barcodes, BC) allowing their identification by sequencing, thus facilitating the design of pool experiments. In this work, the pooled strains were subjected to FLC selection in the presence of doxycycline (Dox). Under these conditions, strains expressing genes mediating tolerance may be favored over those with wild type characteristics.

Prior to selection experiments with this collection of pooled strains, optimal OE conditions had to be determined. We chose to grow the *C. albicans* OE collection in RPMI medium to be consistent with EUCAST culture conditions. It was necessary to determine at which Dox concentration a significant OE could be detected. To address this question, a Dox-dependent *Gaussia princeps* luciferase (*gLUC*) reporter strain (CEC3083) was used. This strain was used to determine the optimal pH and Dox concentration for the pool enrichment assay (Supplementary Figure S2). The luminescence signal became saturated at 100 μg/ml Dox and at both pH 7 and 7.5. The medium pH also influenced the level of detected luminescence. At pH 4.5, the signal was at least 10-fold lower compared to the other pH conditions and independently of the Dox concentration. Luminescence signals resulting from Dox addition appeared similar between pHs 6.0, 7.0, and 7.5. Thus, with these observations, pH 6.0 and 7.0 seemed optimal for gene OE. It is also known that Dox acts synergistically with FLC, converting this drug into a fungicidal rather than fungistatic drug. This synergistic effect is due to the ability of Dox to chelate iron but can be reversed by addition of iron (FeCl_3_) in the medium (Fiori and Van Dijck, 2012).

### Enrichment in Resistant/Tolerant OE Strains in Pool Assay and Single Profiling

**FIGURE 3:**
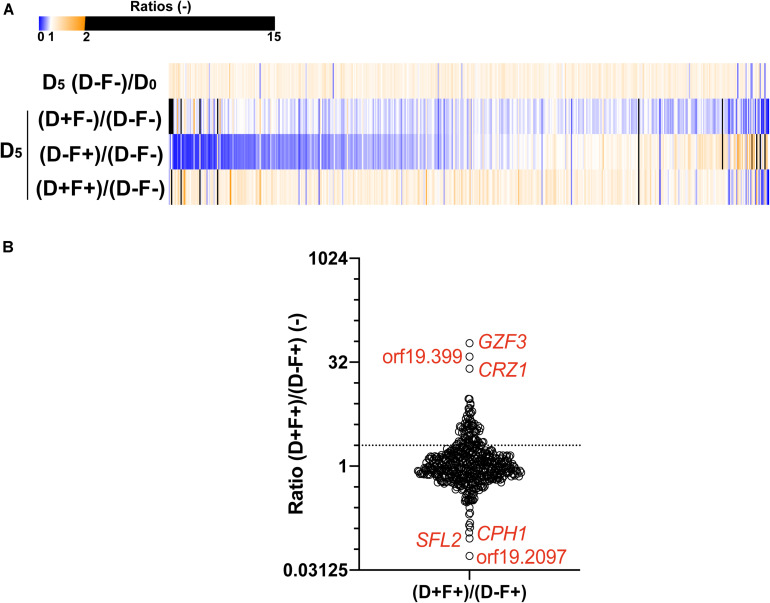
Comparisons of the population of the 572-Tet-inducible OE strains collection pool after 5 days of enrichment. **(A)** The heatmap represents the comparison between the cultures at day 5 (D_5_) and the initial population (D_0_), in presence (F+) or absence (F–) of FLC (32 μg/ml), and in the presence (D+) or absence (D–) of 100 μg/ml Dox. Each line represents the mean relative count ratio of biological duplicates. The heatmap is filtered based on the [D_5_ (D+F+)/D_5_ (D–F+)] comparison. **(B)** Enrichment of specific Tet-dependent OE strains in the presence of FLC with and without Dox. The dotted line represents a 2-fold threshold enrichment between FLC exposed cells in the absence and presence of Dox. *Y*-axis is in log2 scale. Detailed data can be found in Supplementary File S2.

In order to identify new mediators of tolerance using the OE collection, an enrichment in tolerant strains from a pool was performed. Briefly, the 572 strains were pooled and grown under strong FLC pressure (32 μg/ml) and in the presence or absence of Dox (100 μg/ml) at pH 6.0. To select tolerant strains, the culture was maintained for 5 days with daily re-inoculation into fresh media to maintain FLC and Dox concentrations. After 5 days of subculture, the fraction of each strain in the population was estimated for each tested condition by sequencing of the BC. To evaluate the effect of this prolonged culture on the evolution of population, the BC population of the fifth day (D_5_) in the absence of Dox and FLC [D_5_ (D−F−)] was compared to the initial population (D_0_). The mean ratio D_5_ (F-D-)/D_0_ measured for all strains was 0.99 (Figure 3A), suggesting that strains were represented in the same proportion at D_5_ and D_0_ and confirming that fluctuations in populations at D_5_ are due to the different growth conditions (Figure 3A, top line). To evaluate the effect of FLC and Dox, the proportion of each strain in the presence of both compounds [D_5_ (D+F+)] was compared to the reference condition [D_5_ (D−F−)] (Figure 3A, second line). Strains were up to 600-fold enriched and 25-fold depleted in the presence of FLC and Dox as compared to the reference (Supplementary File S2). Comparison of the population in the presence of FLC only [D_5_ (D-F+)] to the reference allowed the identification of intrinsically resistant/tolerant strains with strains up to 900-fold enriched in presence of FLC and highly sensitive strains which were up to 15-fold depleted (Figure 3A, third line, Supplementary File S2). To determine the effect of Dox in the absence of FLC on the population, the population in presence of Dox only [D_5_ (D+F−)] was compared to the reference condition. Here, strains were up to 15-fold enriched and up to 500-fold depleted at D_5_ (D+F−), indicating a slight effect of Dox on the population which is probably linked to the non-tightly regulated OE system (Figure 3A, fourth line, Supplementary File S2). Finally, the effect of Dox in presence of FLC was estimated by comparing the population D_5_ (D+F+) to the population D_5_ (D−F+) (Figure 3B, bottom line). This last comparison is expected to give valuable insight into the genes that mediate a selective growth advantage in the population when overexpressed in presence of FLC. In this comparison 78 strains were at least 2-fold enriched and 39 strains 2-fold depleted when their respective genes were overexpressed under strong FLC pressure (dotted line of Figure 3B and Supplementary File S2). Among them, the three top strains were those overexpressing *GZF3*, orf19.399, and *CRZ1* (60-fold, 38-fold, and 25-fold enriched, respectively) (Table 1). On the other hand, the three strains which were the least represented in the pool were the strains overexpressing orf19.2097, *SFL2* and *CPH1* (18-fold, 11-fold, and 9-fold depleted, respectively) (Figure 3B and Table 1).

**TABLE 1:** Top 10 enriched and depleted strains after 5 days of subculture.

	orf n°	Gene name	(D−F−)^1^/D_0_^2^	D_5_^2^ (D+F+)/(D−F+)	Category
Top 10 enriched strains	orf19.2842	*GZF3*	1.07	60.60	Transcription factor
	orf19.399	NA^3^	1.08	38.63	Protein kinase
	orf19.7359	*CRZ1*	1.07	25.87	Transcription factor
	orf19.5157	NA	1.18	9.43	Protein phosphatase
	orf19.5335	*SGS1*	1.36	9.36	DNA replication/recombination/repair
	orf19.3715	*ASF1*	0.96	8.07	DNA replication/recombination/repair
	orf19.2324	*UBA4*	1.25	7.73	Protein phosphatase
	orf19.4166	*ZCF21*	1.14	6.93	Transcription factor
	orf19.2320	NA	1.41	6.86	Protein kinase
	orf19.2907	*PGA42*	1.14	6.21	Cell wall gene
Top 10 depleted strains	orf19.217	NA	0.81	0.30	Transcription factor
	orf19.4979	*KNS1*	1.38	0.25	Protein kinase
	orf19.3300	*ZPR1*	0.43	0.21	Transcription factor
	orf19.1189	NA	0.99	0.20	DNA replication/recombination/repair
	orf19.6936	*RAD53*	0.99	0.15	Protein kinase
	orf19.7208	*CSK1*	0.97	0.14	Protein kinase
	orf19.5032	*SIM1*	0.26	0.13	Various
	orf19.4433	*CPH1*	0.86	0.11	Transcription factor
	orf19.3969	*SFL2*	0.87	0.09	Transcription factor
	orf19.2097	NA	0.83	0.05	Transcription factor

*The effect of 5 days of subculture [D_5_ (D−/F−)/D_0_] and the effect of the gene OE in presence of FLC [comparison with the initial pool population (D_5_ (D+/F+)/(D−/F+))] on the pooled population is represented as the fold change of the presence of a strain in the population. ^1^D, doxycycline; F, fluconazole, +/-, presence/absence of the drug. ^2^D_0_, initial population (Day 0); D_5_, population after 5 days on enrichment. ^3^Not available.*

**FIGURE 4:**
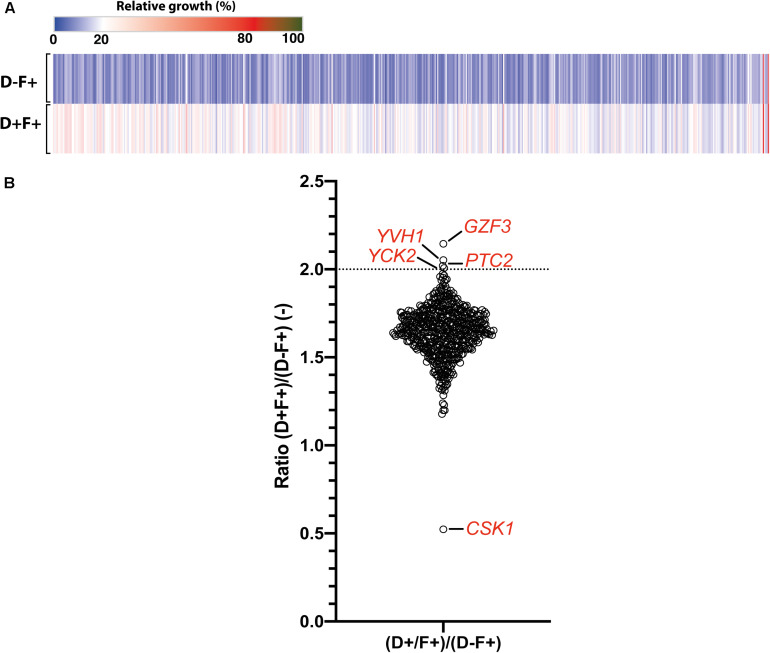
Single strain tolerance profiling. **(A)** Heatmap representing the relative growth of the 572 Tet-inducible OE BC strains collection at pH 6, in presence of 32 μg/ml FLC pressure (F+) and in presence (D+) or absence (D–) of 100 μg/ml Dox. Each line represents the mean relative growth of biological triplicates. **(B)** Effect of Dox induction upon FLC tolerance represented as the growth ratio between the condition with and without Dox [(D+F+)/(D–F+)]. Specific genes above a ratio of 2-fold relative growth increase (dotted line) and below a ration of 1.0 are indicated. Detailed data can be found in Supplementary File S2.

To avoid a bias caused by growth in pools and to confirm the candidate genes identified in the pool enrichment procedure, the tolerance profile of each strain in the collection was tested individually (Figure 4). Growth in the presence of FLC and Dox (D+F+) and in the presence of FLC only (D−F+) were compared to growth in the F−D− control condition. Relative growth in the presence of Dox and FLC (D+F+) ranged from 14 to 67% as compared to the F−D− control condition, while relative growths under FLC pressure only (D−F+) was comprised between 8 and 65% (Supplementary File S5). In order to select tolerant/resistant strains, isolates with a relative growth at least 2-fold higher in the presence of Dox and FLC (D+F+) than in presence of FLC only (D−F+) were selected (Figure 4A and Supplementary File S2). Interestingly, unlike results obtained from the pool enrichment assay (78 strains at least 2-fold enriched), only four strains exhibited a 2-fold increase of growth in presence of FLC and Dox in the single strain assay (Figure 4B). This suggests a pool effect in the pool enrichment assay, which may be due to the duration of the pool experiment (5 days). These four strains included those overexpressing *GZF3* (2.14-fold growth increase), *YVH1* (2.05-fold increase), *PTC2* (2.02-fold increase), and *YCK2* (2.01-fold increase) (Figure 4B and Table 2). On the other hand, no strains exhibited relative growth at least 2-fold reduced when Dox was added. Indeed, only the strain overexpressing *CSK1* showed a decreased growth in presence of Dox (0.52-fold decrease), but this growth depletion was due to an abnormal high growth (65% of the growth control) when grown in presence of FLC only (Figure 4). The difficulty to observe growth reduction upon Dox induction in presence of FLC might be due to the already low tolerance level of the parental CEC161 strain used to build the collection. Interestingly, the *GZF3* OE strain was the best hit in both pooled and single assays approaches, making it an engaging potential mediator of FLC tolerance. Using results from both the enrichment and the single strains assays, a total of twelve strains were selected for further characterization of their FLC susceptibility and tolerance profile including the strains overexpressing *GZF3*, *CRZ1*, *PTC2*, *YVH1*, *YCK2*, orf19.399, orf19.5157, orf19.2320, *SGS1*, *ASF1*, *UBA4*, and *ZCF21*.

**TABLE 2:** Listing of the 10 top-hit strains showing the highest and lowest growth ratio in the single strain FLC tolerance assay.

	orf n°	Gene name	Relative growth (% GC)^1^		Fold change (−)	
			D−F+^2^	D+F+	(D+F+)/(D−/F+)	Category
Top 10 strains	orf19.2842	*GZF3*	12.44	26.35	2.14	Transcription factor
with increased	orf19.4401	*YVH1*	9.67	19.82	2.05	Protein phosphatase
tolerance	orf19.2538	*PTC2*	12.75	25.43	2.02	Protein phosphatase
	orf19.7001	*YCK2*	14.03	28.24	2.01	Protein kinase
	orf19.3356	*ESP1*	11.32	22.34	1.97	DNA replication/recombination/repair
	orf19.5144	*PGA28*	11.61	22.87	1.97	Cell wall gene
	orf19.6792	*RRD1*	13.17	25.83	1.96	Protein phosphatase
	orf19.799	*STE4*	13.06	25.42	1.95	Other signaling comp
	orf19.5636	*RBT5*	10.36	20.09	1.94	Cell wall gene
	orf19.5257	*LCB4*	14.57	27.58	1.94	Various
10 strains	orf19.3199	*PIKA*	13.76	17.56	1.32	Various
ranking last in	orf19.3294	*MBF1*	13.47	17.61	1.31	Transcription factor
the TI screen	orf19.1759	*PHO23*	18.91	24.34	1.31	Transcription factor
	orf19.4377	*KRE1*	16.48	20.01	1.28	Cell wall gene
	orf19.4473	NA^3^	55.66	67.60	1.24	Uncharacterized
	orf19.7652	*CKA1*	17.28	19.70	1.23	Protein kinase
	orf19.6926	*CSC25*	13.31	15.76	1.20	Protein kinase
	orf19.3589	*SPO11*	14.05	16.82	1.20	DNA replication/recombination/repair
	orf19.7473	NA	53.59	63.65	1.18	DNA replication/recombination/repair
	orf19.7208	*CSK1*	65.37	34.08	0.52	Protein kinase

*^1^% of the drug free growth control. ^2^D, doxycycline; F, fluconazole, +/−, presence/absence of the drug. ^3^NA, not available.*

### Positive Regulators of FLC Tolerance

**FIGURE 5:**
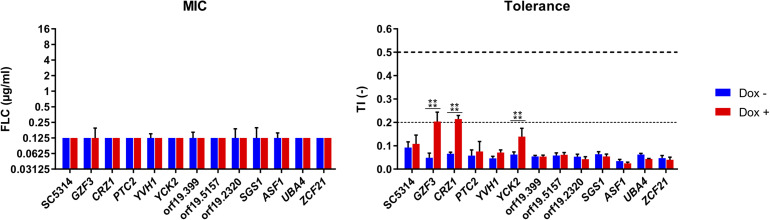
Susceptibility profiles of selected Tet-inducible strains in the presence Dox. Susceptibility profiles were performed at pH 6, in a range of FLC concentrations (0–16 μg/ml), in the presence (Dox+) or absence (Dox–) of doxycycline at 100 μg/ml and complemented with 1 mM FeCl_3_. FLC susceptibility was presented by their modal MIC value (left panel) and TIs at 8 μg/ml FLC (right panel). Standard deviations of the MIC values were calculated based on the mean MIC of each sample. The horizontal dashed line represents the lower threshold of tolerance (TI = 0.2) and the limit of susceptibility (TI = 0.5) as defined by EUCAST. Each bar is representative of biological triplicates. Statistical significance was determined using Sidak’s multiple comparison tests (Two-way ANOVA, Confidence interval = 95 %). *P*-values: (****) ≤ 0.0001.

Given that the genetic background of the *C. albicans* BWP17-derived strain used in the previous screenings exhibits low FLC tolerance, this made difficult to address potential negative regulators of FLC tolerance. We rather focused on positive putative positive tolerance regulators. The 12 selected isolates of the OE collection including the wild type SC5314 were re-tested individually to establish their FLC susceptibility profiles at pH 6.0 in the presence of Dox (Figure 5). At this Dox concentration, all strains exhibited similar MIC independently of Dox induction, with all MICs remaining at around 0.125 μg/ml FLC. FLC tolerance was assessed by TIs at the fixed FLC concentration of 8 μg/ml and by comparing TIs in the absence and presence of Dox. In this assay, significant increase of TIs in the presence of Dox was only obtained in the strains overexpressing *GZF3* (TI = 0.048–0.2), *CRZ1* (TI = 0.07–0.21) and *YCK2* (TI = 0.06–0.14). *CRZ1* is a calcineurin-regulated transcription factor already identified to play a role in FLC tolerance by Onyewu et al. (2004) and Karababa et al. (2006) and recently by Rosenberg et al. (2018). *GZF3* is a putative GATA-type transcription factor, of which the *Saccharomyces cerevisiae* ortholog negatively regulates nitrogen catabolic gene expression (Soussi-Boudekou et al., 1997). *YCK2* encodes a plasma membrane protein similar to the *S. cerevisiae* casein kinase I, which is required for the membrane trafficking of Pdr5 (the ortholog of the multidrug transporter *CDR1* in *S. cerevisiae*) to the cell surfaces (Decottignies et al., 1999). The re-identification of *CRZ1* here underscores that the selection of FLC mediators by the undertaken experimental approaches is feasible, even if limitations have to be considered (see below). We focused next only on *CRZ1* and *GZF3*, since the Dox-dependent overexpression of these genes in *C. albicans* reached the threshold of tolerance (TI ≥ 0.2) that we previously defined for clinical isolates.

### *CRZ1* and *GZF3* OE in Deletion Mutants

**FIGURE 6:**
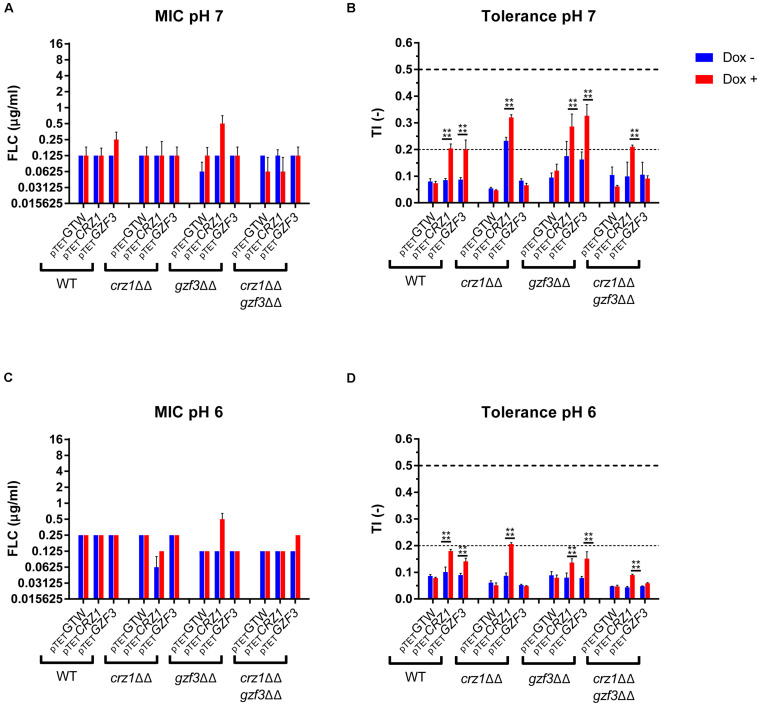
Susceptibility profile of the *CRZ1* and *GZF3* mutants overexpressing either *CRZ1*, *GZF3*. **(A,C)** Susceptibility to FLC is represented as the modal MIC value with standard deviation calculated on the mean MIC of all replicates. **(B,D)** Tolerance profile of the mutant strains. The mean TI and standard deviations are represented. Horizontal dashed lines represent the lower threshold of tolerance (TI = 2), and the upper limit of susceptibility (TI = 0.5) as described by EUCAST. Each data set represents the mean (or mode) of biological duplicates. Statistical significances **(B,D)** were estimated using Sidak’s multiple comparison tests (two-way ANOVA, 95% confidence interval) between Dox– and Dox+ conditions in the same mutants set. *P*-values: (****) ≤ 0.0001. Supplementary Table S1 lists correspondence between genotype and strain number.

In order to better understand the mechanisms behind tolerance and the potential relation between *CRZ1* and *GZF3*, each gene was overexpressed in single and double mutants. For this purpose, a *crz1*Δ/Δ, *gzf3*Δ/Δ double mutant (EDY28-2, Supplementary Table S1) was constructed using the *crz1*Δ/Δ mutant from the Homann’s deletion mutant collection (Homann et al., 2009). The OE system for either *CRZ1* or *GZF3* was then transformed in this double mutant, in both Homann’s *crz1*Δ/Δ and *gzf3*Δ/Δ single mutants as well as in the parental WT strain of the Homann’s collection. A control plasmid (CIp10-P_TET_-GTW, Supplementary Table S1) was also transformed in each strain as control of the OE system. The FLC susceptibility profiles of these strains were then established in presence or absence of Dox and FLC to assess their respective MICs and TIs (Figures 6A–D). These susceptibility assays were performed at pHs 6.0 and 7.0.

Overall, all strains exhibited similar MIC values in presence or absence of Dox. The MIC for all tested strains at pH 7.0 was equal to 0.0625 μg/ml FLC with negligible variation. The *gzf3*Δ/Δ mutant overexpressing *CRZ1* showed the highest increase in MIC value, reaching 0.25 μg/ml FLC in presence of Dox (Figures 6A,C). Globally, the susceptibility profile remained the same at pH 6.0. With regards to FLC tolerance, all strains showed a basal TI under the lower tolerance threshold (i.e., TI = 0.2) at both pH values in absence of Dox. Consistent with observations of the pool enrichment assay, *CRZ1* and *GZF3* OE resulted in a significant increase of the TI in a WT background. In *gzf3Δ/Δ* mutants, significant increase in TI could be observed at both pHs under *CRZ1* and *GZF3* OE. Indeed, TI increased from 0.17 to 0.28 and 0.16 to 0.31 at pH 7.0 and from 0.07 to 0.13 and 0.07 to 0.14 at pH 6.0 for *CRZ1* and *GZF3* OE, respectively (Figures 6B,D). On the other hand, only *CRZ1* OE resulted in an increased in tolerance in the double *crz1*Δ/Δ, *gzf3*Δ/Δ mutant. No effect of *GZF3* OE on tolerance could be observed in the *crz1Δ/Δ* mutant as well as in the double *crz1*Δ/Δ, *gzf3*Δ/Δ mutant (Figures 6B,D). However, *CRZ1* OE in these mutant backgrounds resulted in increased tolerance at pH 7.0 and 6.0, respectively (Figures 6B,D).

**FIGURE 7:**
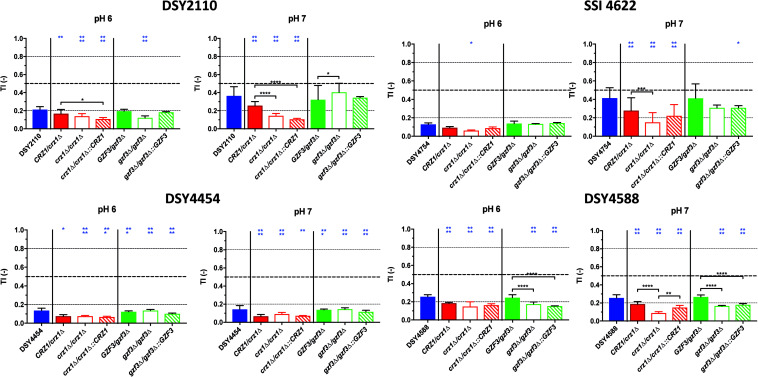
Tolerance levels of the different deletion mutants for both *CRZ1* and *GZF3* in the DSY2110, DSY4454, SSI 4622, and DSY4588 genetic backgrounds at pHs 6.0 and 7.0. Each bar corresponds to at least biological duplicates. Statistical significance was calculated using Turkey multiple comparison tests (95% confidence interval). *P*-values: 0.1234 (ns), ≤0.0332 (*), ≤0.0021 (**), ≤0.0002 (***), ≤0.0001 (****). Blue stars correspond to the comparison with the parental clinical isolates (blue bars). Black stars correspond to the comparisons between related mutant strains. Supplementary Table S1 lists correspondence between genotype and strain number.

To summarize, our results indicate that overexpressing *CRZ1* results in increased FLC tolerance in all mutant backgrounds but, on the other hand, *GZF3* OE increases tolerance only when *CRZ1* was present. These results suggest that *CRZ1* controls the expression of one or more intermediary proteins or cofactors necessary for *GZF3* activity, which in its turn could activate other regulators of tolerance. Taking the hypothesis that *CRZ1* and *GZF3* may be part of a same pathway, our results indicate that *GZF3* may act upstream of *CRZ1*.

### *CRZ1 a*nd *GZF3* Deletions in Tolerant Clinical Isolates

To confirm the role of *CRZ1* and *GZF3* in promoting tolerance to fluconazole not only in laboratory strains, either one or both allele of each gene was deleted in the *C. albicans* clinical isolates DSY2110, DSY4454, SSI 4622 and DSY4588, already known to exhibit different level of FLC tolerance (Supplementary Figure S3). Revertant strains were constructed by reinsertion of *CRZ1* and *GZF3* alleles from the reference isolate SC5314. The FLC susceptibility profiles of each mutant and associated parental clinical isolates were assessed at pHs 7.5, 7.0, 6.0, and 4.5 and their MIC values and TIs extracted for comparisons. Globally, no differences could be observed between the MIC values of the original clinical isolates and their respective mutants, with all MICs standing below the FLC clinical breakpoint (CBP) of 4 μg/ml (Supplementary Figure S4).

When inspecting variations of FLC tolerance in the generated mutants and revertants, a general trend was that deletion of *CRZ1* reduced tolerance levels in all tested strains (for convenience only data from pH 6.0 and 7.0 measurements are shown in Figure 7, all pHs are shown in Supplementary Figure S5). Only for strain DSY4488, the deletion of *GZF3* produced little but significant reduction of FLC tolerance at pHs 6.0 and 7.0 as compared to the initial clinical strain. Reversion of the gene deletions by wild type *CRZ1* and *GZF3* alleles from SC5413 at their genomic loci was not always resulting in initial tolerance levels. For example, *CRZ1* restoration in the background of the *crz1*Δ/Δ mutant of DSY2110 did not revert tolerance to the levels of the wild type clinical strains as well as hetero- and homozygous *CRZ1* mutants. We confirmed by another phenotypic test (SDS susceptibility, Supplementary Figure S6) that the *CRZ1* revertant could complement the mutant phenotypic defects, showing that the re-introduced *CRZ1* allele was still functional. Taken together, the results of *CRZ1* and/or *GZF3* deletions and reversions revealed that *CRZ1* was the factor that most prominently altered FLC tolerance of the clinical stains. The results were dependent on the tested pHs and on the genetic background of the isolates.

### Transcriptomic Imprints of FLC Response in FLC-Tolerant Clinical Isolates

As shown earlier, clinical strains exhibit several degrees of FLC tolerance. One alternative approach to address the mechanisms behind the development of FLC tolerance is to compare transcriptional profiles between strains exhibiting different FLC tolerance levels. It was of interest to address the transcriptional profile of such strain in the presence of FLC under the conditions in which tolerance was tested. Given that the best option of comparisons is between related isolates, we inspected our strain collection for related strains exhibiting different FLC tolerance profiles. The chosen strains were originating from a group of related strains including DSY4454, DSY4452 and DSY4588. These strains were recovered from a patient suffering of endocarditis which was treated for an extensive period with FLC (8 years). DSY4454 was the earliest strain recovered (year 2005) from the patient, while DSY4588 was the latest (year 2013). Patient and sampling details are given in Supplementary File S6 and FLC tolerance profiles in Supplementary Figure S7. DSY4454 exhibited low TIs at all pHs (except at pH 4.5), while DSY4452 and DSY4588 were showing TIs above the 0.2 threshold value at neutral pHs. The relationship between strains was investigated with Multi Locus Sequence Typing (MLST, Supplementary File S6) (Gow et al., 2003). A single loss of heterozygosity (LOH) event was detected in the *SYA1* allele of isolate DSY4588 as compared to the other strains, thus suggesting that micro-evolution between the strains could have occurred. However, the MLST results indicated that the strains were from the same type, thus suggesting a strong genetic relationship between them.

**FIGURE 8:**
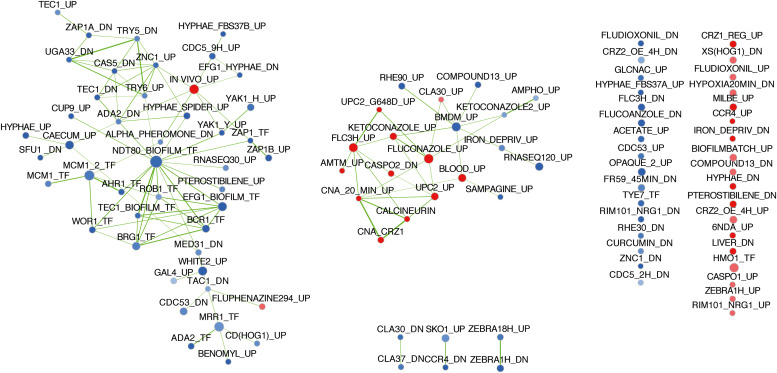
GSEA enrichment map of FLC regulated genes in clinical isolate DSY4452. Analysis parameters were as follows: norm, meandiv; scoring_scheme, weighted; set_min, 15; nperm, 1000; set_max, 500. GSEA results were uploaded into Cytoscape 3.0 with the following parameters: *P*-value cutoff, 0.01; FDR *q*-value, 0.05. Red nodes represent enriched gene lists in upregulated genes from the GSEA. Blue nodes represent enriched gene lists in downregulated genes from the GSEA. Nodes are connected by edges when overlaps exist between nodes. The size of nodes reflects the total number of genes that are connected by edges to neighboring nodes. Edge thickness reflects the level of confidence between nodes.

RNAseq analysis was carried out as described in section “Materials and Methods.” The isolate SC5314 was included in these RNAseq analysis as a control isolate exhibiting low level of tolerance (Supplementary Figure S7). Gene expression changes were extracted by pairwise comparison between conditions with and without FLC. The data were filtered for significance (False Discovery Rate, FDR ≤ 0.05) and for expression change (≥2-fold) between drug-exposed and non-exposed conditions (Supplementary File S7). The filtered data are summarized in Table 3. The FLC-regulated genes from the clinical strains DSY4452, DSY4454, DSY4588 and SC5314 were next subjected to Gene Set Enrichment Analysis (GSEA) using a database of gene list originating from 307 published studies (Supplementary File S8). This database includes conditions in which (i) *C. albicans* was exposed to different antifungal agents, (ii) *C. albicans* mutants were exposed to different growth conditions, (iii) *C. albicans* was exposed to host cells and (iv) *C. albicans* was grown *in vitro* under conditions mimicking host environments. The GSEA list also contains *C. albicans* genes with binding sites for several transcription factors. In general, the used conditions are divided into lists of up-and down-regulated genes. The software compares a query list of up- and down-regulated genes with existing gene lists and identifies those that overlap through statistical evaluation. A network of associated gene lists can be formed using visualization software such as Cytoscape (GSEA enrichment map) depending on the overlaps between conditions. These data are summarized in Figure 8 (for simplicity only the GSEA map for DSY4452 is shown).

**TABLE 3:** Genes regulated by FLC in the clinical strains.

Isolate	FLC upregulated genes	FLC downregulated genes
DSY4454	484	579
DSY4452	370	471
DSY4588	451	262
SC5314	824	817

The GSEA map for DSY4452 highlights several features which can be also observed in the maps of the other isolates (Supplementary Figure S8). First, the genes regulated by FLC in the clinical isolates overlapped as expected with published datasets in which *C. albicans* was exposed to FLC in different growth conditions. The connected nodes “FLUCONAZOLE_UP” and “FLC3H_UP” were overlapping with most FLC-upregulated genes in the clinical strains. “FLUCONAZOLE_UP” data originate from a study (Vasicek et al., 2014) in which *C. albicans* was exposed for 6 h in the presence of 10 μg/ml FLC in YEPD at 30°C. “FLC3H_UP” data are from a study (Keller et al., 2015) in which *C. albicans* was treated with 0.5 μg/ml FLC for 3 h in RPMI medium at 37°C. All these experiments were performed under agitation in liquid cultures. Considering that most FLC-downregulated genes are also found in the nodes “KETOCONAZOLE_DN,” “FLC3H-DN,” and “FLUCONAZOLE_DN,” the results obtained here are consistent with already published transcriptional data of FLC-exposed cells.

When looking at FLC-upregulated genes, the GSEA data of DSY4452 contained clusters of nodes related to the calcineurin pathway (CALCINEURIN,” “CNA_CRZ1” and “CNA 20 min_up”). Strikingly, these nodes were connected to those originating from FLC-exposed conditions (“FLUCONAZOLE_UP,” “KETCONAZOLE_UP,” “FLC3H_UP”) and from nodes of transcription factors controlling sterol homeostasis (“UPC2_UP”). These networks, containing genes positively regulated by FLC, were also associated with a node comprising genes upregulated by *UPC2* with a gain of function mutation (“UPC2_G648D_UP”) (Dunkel et al., 2008). This underlines the importance of this transcription factor in azole response. Taken together, these data suggest that the calcineurin pathway is connected to the response of *C. albicans* to FLC, which is consistent with the connection made above between *CRZ1* and FLC tolerance.

In order to identity potential mediators of tolerance in the set of clinical strains, the expression of which could vary between wild type (DSY4454) and FLC-tolerant isolates (DSY4452 and DSY4588), the ratios of FLC-regulated genes were determined between pairs of isolates. Expression ratios were first calculated between DSY4452 and DSY4454 and between DSY4588 and DSY4454 (Supplementary File S8). Significant fold-changes were selected between these conditions (*p*-values ≤ 0.05) and the data were filtered with a 2-fold change in expression as cut-off. The expression ratio between DSY4452 and DSY4454 was changed by more than 1.5-fold for 41 genes (Supplementary File S9). Interestingly, 12 out these 41 genes were regulated by calcineurin and/or *CRZ1*. Moreover, the expression ratios of these 41 genes between DSY4588 and DSY4454 were above the 1.5-fold threshold. These results suggest a conservation of the FLC response between the tolerant strains DSY4452 and DSY4588 as compared to DSY4454. Intriguingly, most of the ratios of the 41 above-mentioned genes between DSY4454 and SC5314, both of which could be considered as “wild type” in terms of FLC tolerance, were below the 1.5-fold threshold. Taken together, the data presented here suggest that the expression of genes that are known to be calcineurin- and/or *CRZ1*-dependent is increased in the FLC-tolerant clinical strains and this indicates that the calcineurin pathway is modified by still uncharacterized factors in these strains.

## Discussion

In this study, we aimed to better define FLC tolerance and to identify FLC tolerance mediators in *C. albicans*. The term of tolerance is too often confounded with the description of drug resistance in published studies. We first addressed adequate methods for the quantification of FCL tolerance in order to facilitate comparisons between different isolates. Standardized microdilution susceptibility assays that include CLSI and EUCAST protocols are widely used to assess antifungal susceptibility of fungi. We took advantage of these established assays to measure FLC tolerance. However, since the EUCAST protocol integrates the use of optical density measurements as readouts, this protocol was chosen as an assay to quantify tolerance in *C. albicans*. In principle, the assay consists in measuring optical density in microdilution plates at a fixed FLC concentration (8 μg/ml) above the CBP MIC threshold of 4 μg/ml FLC and to express growth as a relative value (from 0 to 1), which we called as “tolerance index” (TI). Recently, an alternative tolerance assay was published that used static growth conditions on disk diffusion assays with agar plates. Growth inside the inhibition zone was quantified and served as a basis to quantify FLC tolerance (Rosenberg et al., 2018).

Using a large collection of *C. albicans* isolates, among which some were genome-sequenced, the measured TIs enabled to split isolates into 3 major categories. The first category included isolates with TI values ≤ 0.2 and were ranked as wild type FLC-susceptible isolates. The second category included isolates with TIs values > 2 and <0.8. These isolates were considered as FLC-susceptible but FLC-tolerant. The third category included isolates with TIs values ≥ 0.8 and included isolates ranked as FLC-resistant. This categorization was consolidated by the fact that most isolates with TI > 0.8 were exhibiting known azole-resistance mechanisms (Figure 2). The TI threshold value of 0.2 for distinction between wild type and tolerant isolates is less obvious to consolidate by the occurrence of specific mechanisms, since tolerance mechanisms are not yet associated with specific gene mutations However, this threshold enables to discard a dominant population of isolates that have wild type characteristics in the query of tolerance mechanisms.

One of the observations made in the tolerance assays is that there was a pH-dependent effect in tolerance profiles. Using a specific subset of isolates, we observed that acidic pH increased FLC tolerance (Figure 1). An opposite tendency (decreasing tolerance with decreasing pH) was reported earlier by Marr et al. (1999) and more recently by Rosenberg et al. (2018). It is not clear as to why our results differ from these published results. The methods used in these studies referred to minimal and complex culture media and to agar diffusion assays, respectively. Thus, one might argue that these assay conditions could have impacted on the obtained results. Since different isolates were used in these different studies, it is difficult to identity the cause of such disagreements. Our own results with RPMI and YEPD media in FLC tolerance assays indicate that the use of YEPD -similar to the medium used by Rosenberg et al. (2018)- results in a drop of FLC tolerance at low pH in some strains, but not all of them, as compared to RPMI (Supplementary Figure S9). This suggests that the medium composition is an important variable in FLC tolerance assays, however, the genetic background of the tested strains may also contribute to this variable. This is in agreement with results of Figure 2 gathering TIs of a large set of strains highlight that the pH dependency of FLC tolerance is a rather strain-dependent attribute, since about 70% of tested isolates (categories 4 and 5) do not exhibit an increase of FLC tolerance at low pH in RPMI. Another study was published by Luna-Tapia et al. (2015) in which a *VSP21* mutant exhibited high tolerance to FLC at both pH 7.0 and pH 3.0. In this work, a CLSI protocol was used which therefore was more closely related to the conditions tested here. Luna-Tapia et al. (2015) demonstrated that tolerance of the *VSP21* mutant was decreased at pH 3.0 compared to neutral conditions. While these data support pH dependency on FLC tolerance, the results used a specific mutant which might trigger pH-specific response and might differ from clinical wild type isolates. Thus, pH dependency of FLC tolerance remains controversial.

We next addressed the identification of FLC tolerance mediators using a forward genetic screen with a pool enrichment approach using a collection of 572 barcoded strains. This strategy used the RPMI medium and growth conditions used to determine tolerance in clinical strains. Each of these strains overexpressed a specific gene under the control of Dox (Chauvel et al., 2012). The same set of pooled strains was used by Znaidi et al. (2018) in a genetic screen performed in a murine model of gastro-intestinal colonization. In this study, the authors used the barcodes to identify strains of interest that were enriched and/or depleted in the animal model in the presence of Dox. Different from the barcode sequencing system used in the present study, Znaidi et al. (2018) used a barcode detection system based on microarrays on which all barcode tags were deposited. In the animal model selection system, only one gene, *CRZ2*, was identified as conferring a selective growth advantage when overexpressed in the animal gut. *CRZ2* encodes a zinc finger transcription factor of the C2H2 family that is similar to the calcineurin target Crz1p, however, it is not involved in calcium signaling (Karababa et al., 2006). In the present study the pooled collection was maintained under strong FLC pressure for 5 days in the presence and absence of Dox. We were first able to identify enriched and depleted isolates in these conditions. The susceptibility profiles of these candidate genes were further investigated in single strain assays to address potential pool effects in the enrichment assay. We observed a poor correlation between the pooled and single strain assays. Among the possible explanations, the repeated subcultures as the duration of the experiment (5 days) in the pooled experiment compared to the single strain assay (1 day) might have created several biases in the selection of FLC-tolerant strains. Even considering these limitations, we came with a list of 12 putative mediators of FLC tolerance by crossing these results of both selection experiments. Only two of them, *CRZ1* and *GZF3*, could be linked to a significant increase of tolerance under Dox induction above the clinical threshold (TI > 0.2). One of these two genes was the calcineurin-regulated transcription factor *CRZ1*, already identified to play a role in FLC tolerance by Onyewu et al. (2004) and Karababa et al. (2006). To act as a transcription factor, Crz1 needs to be translocated to the nucleus. This is achieved by dephosphorylation via calcineurin, which is known to act as a protein phosphatase (Karababa et al., 2006). In this work, we showed that *CRZ1* overexpression is triggering FLC tolerance, which may be achieved by transcriptional activation and thus a shift in the balance toward dephosphorylated Crz1. The relationship between *CRZ1* overexpression and Crz1 dephosphorylation has not been tested here but needs to be addressed in future investigations. The other identified gene was the putative GATA-type transcription factor *GZF3*, of which the *S. cerevisiae* ortholog negatively regulates nitrogen catabolic gene expression (Soussi-Boudekou et al., 1997). Little is known about *GZF3* function in *C. albicans*, but it was shown to be induced, via Cap1, in oxidative stress conditions (Wang et al., 2006). Most of the information on *GZF3* come from Homann’s and Noble’s phenotypic screens (Homann et al., 2009; Noble et al., 2010). In their works, the homozygous *gzf3*Δ/Δ mutant resulted in abnormal growth on Spider medium and decreased competitive fitness in a mice systemic infection model. Homann et al. (2009) also observed an increased susceptibility to heat, FLC, lithium chloride, copper and rapamycin. However, no genetic clues could explain these phenotypes so far. To our knowledge, it is the first time here that *GZF3* has been linked to an increase of FLC tolerance when overexpressed in *C. albicans*. A third gene, *YCK2*, was also identified. This gene encodes a plasma membrane protein similar to the *S. cerevisiae* casein kinase I, which is required for the membrane trafficking of Pdr5 (the ortholog of the multidrug transporter *CDR1* in *S. cerevisiae*) to the cell surfaces (Decottignies et al., 1999). *YCK2* overexpression significantly increased FLC tolerance. However, it did not reach the threshold of tolerance (TI < 0.2) defined for clinical isolates. This gene remains, however, a good candidate for future investigations on molecular mechanisms behind FLC tolerance. Interestingly, *YCK2* was showed recently as interfering with caspofungin resistance (Caplan et al., 2020) and was shown earlier to be involved in response to FLC (Jung et al., 2017). The single strain assay also confirmed our pool enrichment protocol to be a valuable approach for the identification of putative positive mediators of FLC tolerance. Additional enrichment experiments using extended OE collections might reveal novel insights into the mechanisms behind this phenotype.

To further characterize the possible interactions existing between *CRZ1* and *GZF3* (since both could mediate FLC tolerance), we observed that *GZF3* significantly increased tolerance when overexpressed, but only when at least a single copy of *CRZ1* was present. Taking the hypothesis that *CRZ1* and *GZF3* may be part of a same pathway, our results suggest that Gzf3 acts upstream of Crz1. Further investigation of Crz1 and Gzf3 target genes via RNA sequencing or chromatin immunoprecipitation may reveal other proteins necessary to increase FLC tolerance as well as highlighting the relation between both TFs. Both *CRZ1* and *GZF3* were also shown to play a role in FLC tolerance in clinical isolates. *CRZ1* deletion significantly reduces FLC tolerance in the four tested clinical strains (Figure 7) to levels below the tolerance threshold (TI < 0.2) of clinical isolates. On the other hand, *GZF3* deletion was able to reduce tolerance levels but only when both alleles were deleted in a specific isolate (DSY4588). Experiments aimed to re-introduce *CRZ1* and *GZF3* in the background of deletion mutants in clinical strains yielded different phenotypic complementation results (Figure 7). For example, *CRZ1* revertant isolates from strains DSY2110 and DSY4454 did not revert to initial parent wild type levels. The reversion of the full initial phenotype may require that two *CRZ1* copies are being re-introduced at their genomic loci. The reversion of the initial phenotype may also require the presence of *CRZ1* alleles originating from the clinical strains and not from SC5314. To confirm this hypothesis, future experiments should introduce the *CRZ1* alleles from the parental clinical strains in their respective homozygote mutants. This last hypothesis suggests that *CRZ1* alleles from these clinical strains could exhibit polymorphisms responsible for the gain of FLC tolerance.

In a recent study published by the group of J. Berman, some mechanistic insights into the basis of FLC tolerance were given (Rosenberg et al., 2018). As suggested by our results, this study highlighted that, using chemically induced phenotypes, the calcineurin pathway was implicated in the development of azole tolerance. The study of Rosenberg et al. (2018) used mutants constructed in laboratory strains (for example *crz1*, *cnb1*) and reduction of trailing growth on disk diffusion assays to support their hypothesis. In addition, this study indicated that the development of tolerance in clinical strains still susceptible to FLC was attributed to distinct subpopulations (Rosenberg et al., 2018). Among other interesting observations, Rosenberg et al. (2018) observed that tolerant strains were more likely to be isolated from FLC-treated patients than others, which suggested that *C. albicans* may adapt to this drug. If azole treatment is prolonged, tolerant isolates may persist and, thus, may cause more severe damage to the host. However, the relationship between occurrence of FLC tolerance and FLC efficacy is still not clearly established in *C. albicans*. A study from Astvad et al. (2018) undertaken with *C. tropicalis* isolates exhibiting different tolerance profiles tested FLC efficacy in systemic mice infection models. The data of this study established that high FLC tolerance compromised the efficacy of FLC in the mice, thus suggesting a clinical relevance of the tolerance phenomenon.

Transcriptomics is a powerful technology to address gene function and to get insights in circuits activated upon environmental perturbations. Chemical stress can induce transcriptional changes reflecting both general (e.g., detoxification pathways) and specific responses of the organism to alteration of one or more biological pathways that are affected by treatment with the chemical (Hughes et al., 2000). While drug exposed conditions can be helpful in revealing biological meaningful data, the use of clinical strains with specific phenotypes is also useful to identify the mechanisms that control these phenotypes. In this work, we addressed the transcript profiling of several isolates in the presence of FLC, some of which exhibit FLC tolerance. The major difference in these experiments as compared to similar published results using *C. albicans* exposed to FLC was that the incubation conditions were mimicking standard susceptibility assays performed under EUCAST recommendations. In these conditions, we showed here that FLC induced a response that included several calcineurin-dependent genes. Such a response profile has not yet been clearly demonstrated in published profiling studies with FLC. Such an association strongly suggests that FLC recruits the calcineurin pathway, which was already showed to be critical for survival of *C. albicans* exposed to FLC (Sanglard et al., 2003).

The clinical isolates used here in transcriptional analysis were related to each other, which was expected to reduce transcriptional noise that can arise from strains exhibiting different genetic backgrounds. In these sequential isolates, DSY4454 was the first recovered isolate from a patient suffering from endocarditis. DSY4454 exhibited relative low TI as compared to the other isolates (DSY4452 and DSY4588). The patient had been treated for several years with FLC until blood cultures were positive for DSY4452 and DSY4588 about 7 and 9 years after the isolation of DSY4454. It is likely that the duration of FLC treatment along these years selected for isolates with genome modifications favoring, among other phenotypes, the development of azole tolerance. Comparative genomics could lead to resolve the molecular basis of azole tolerance in clinical isolates. This type of approach may be rendered difficult due to the likely genome divergence that evolved during the years of persistence of the strains in the host. Our data revealed that several genes of the calcineurin pathway were upregulated as compared to the initial isolate (DSY4454). Currently the genetic basis for this modification is unknown. In our transcript profiling data, *CRZ1* was more upregulated in the tolerant isolates as compared to wild type isolates (by about 2-fold between DSY4452 and DSY4454 and between DSY4588 and DSY4454, Supplementary File S7). As mentioned earlier in this work, *CRZ1* overexpression mediated by an artificial regulatable promoter is enough to induce FLC tolerance in *C. albicans*. *CRZ1* expression changes could therefore be taken as a candidate marker for azole tolerance. Interestingly, a recent study revealed that the copy number of *CRZ1* was correlated with the occurrence of FCL tolerance in *C. albicans* isolates exposed to FLC. Assuming that *CRZ1* gene copy number may be correlated with increased expression, this study corroborates with our observations (Todd and Selmecki, 2020). How *CRZ1* overexpression mediates azole tolerance is still unknown. No detailed information currently exists on *CRZ1* target genes that could ultimately be responsible for azole tolerance. Identification of *CRZ1* target genes that could mediate azole tolerance needs to be systematically performed. In conclusion, even if the present results converge to the involvement of the calcineurin pathway in the development of azole tolerance, additional studies are needed to resolve the molecular details behind this phenomenon.

## Data Availability Statement

The datasets presented in this study can be found in online repositories. The names of the repository/repositories and accession number(s) can be found in the article/Supplementary Material.

## Author Contributions

ED wrote the manuscript and performed experiments. LB, ET, MP, CP, and FM performed experiments. SZ, MC, ML and M-EB provided material. Cd’E designed strategies. DS designed strategies and wrote the manuscript. All authors contributed to the article and approved the submitted version.

## Conflict of Interest

ED is employed by Resistcell AG. CP is employed by Abionic SA. FM is employed by Philip Morris Product SA. The remaining authors declare that the research was conducted in the absence of any commercial or financial relationships that could be construed as a potential conflict of interest.

## Footnotes

1 https://github.com/dsanglard/Barcode-quantification-2.git

2 http://www.R-project.org/

3 https://software.broadinstitute.org/morpheus
