# Supplementary material for: Identification and Characterization of Mediators of Fluconazole Tolerance in Candida albicans
Source: Front Microbiol. 2020 Nov 11;11:591140. doi: 10.3389/fmicb.2020.591140 (PMC7686038; doi:10.3389/fmicb.2020.591140)
Supplement: Supplementary file 10 [file Presentation_1.PPTX]

## Slide 1
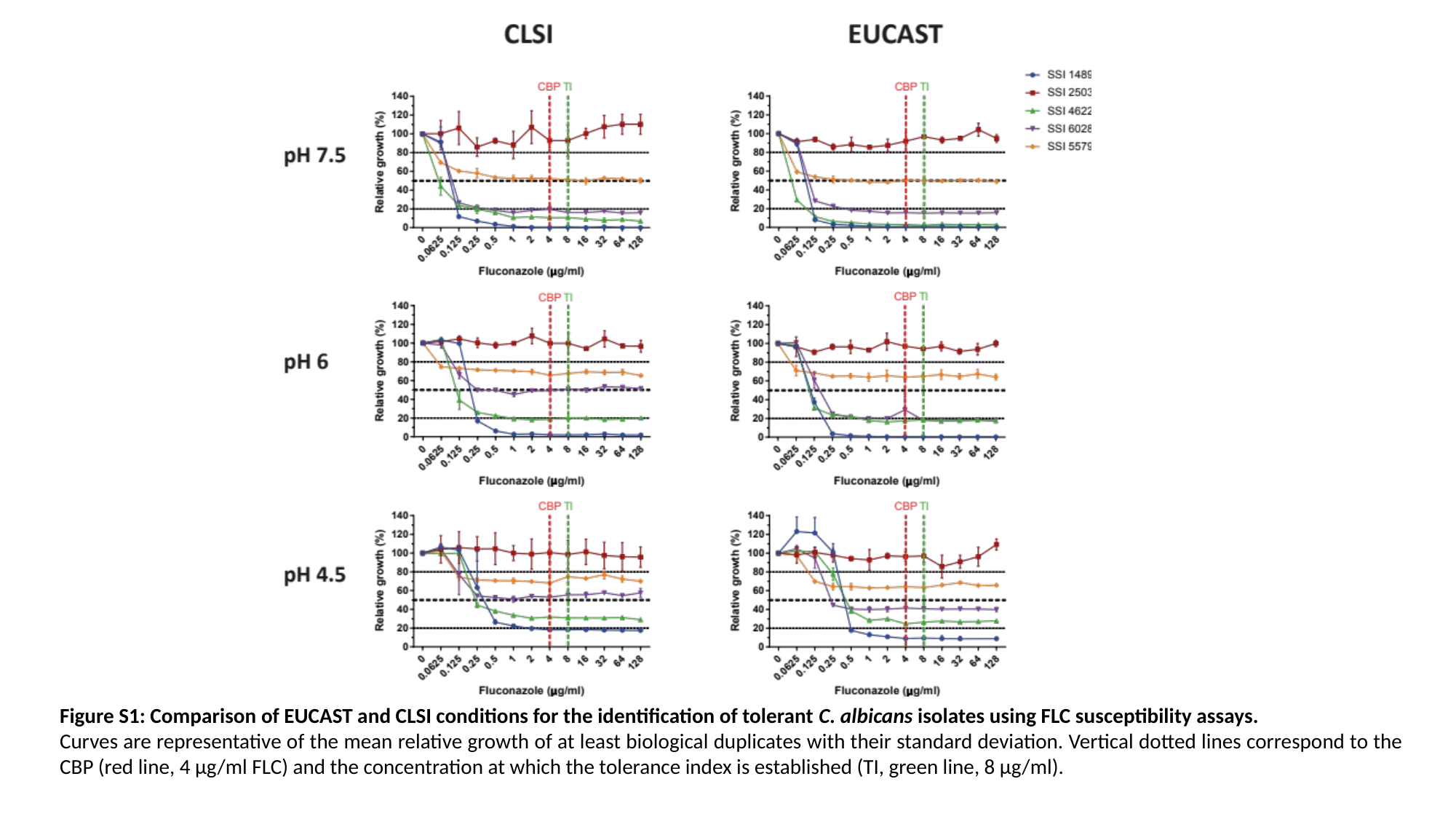

Figure S1: Comparison of EUCAST and CLSI conditions for the identification of tolerant C. albicans isolates using FLC susceptibility assays.
Curves are representative of the mean relative growth of at least biological duplicates with their standard deviation. Vertical dotted lines correspond to the CBP (red line, 4 µg/ml FLC) and the concentration at which the tolerance index is established (TI, green line, 8 µg/ml).

## Slide 2
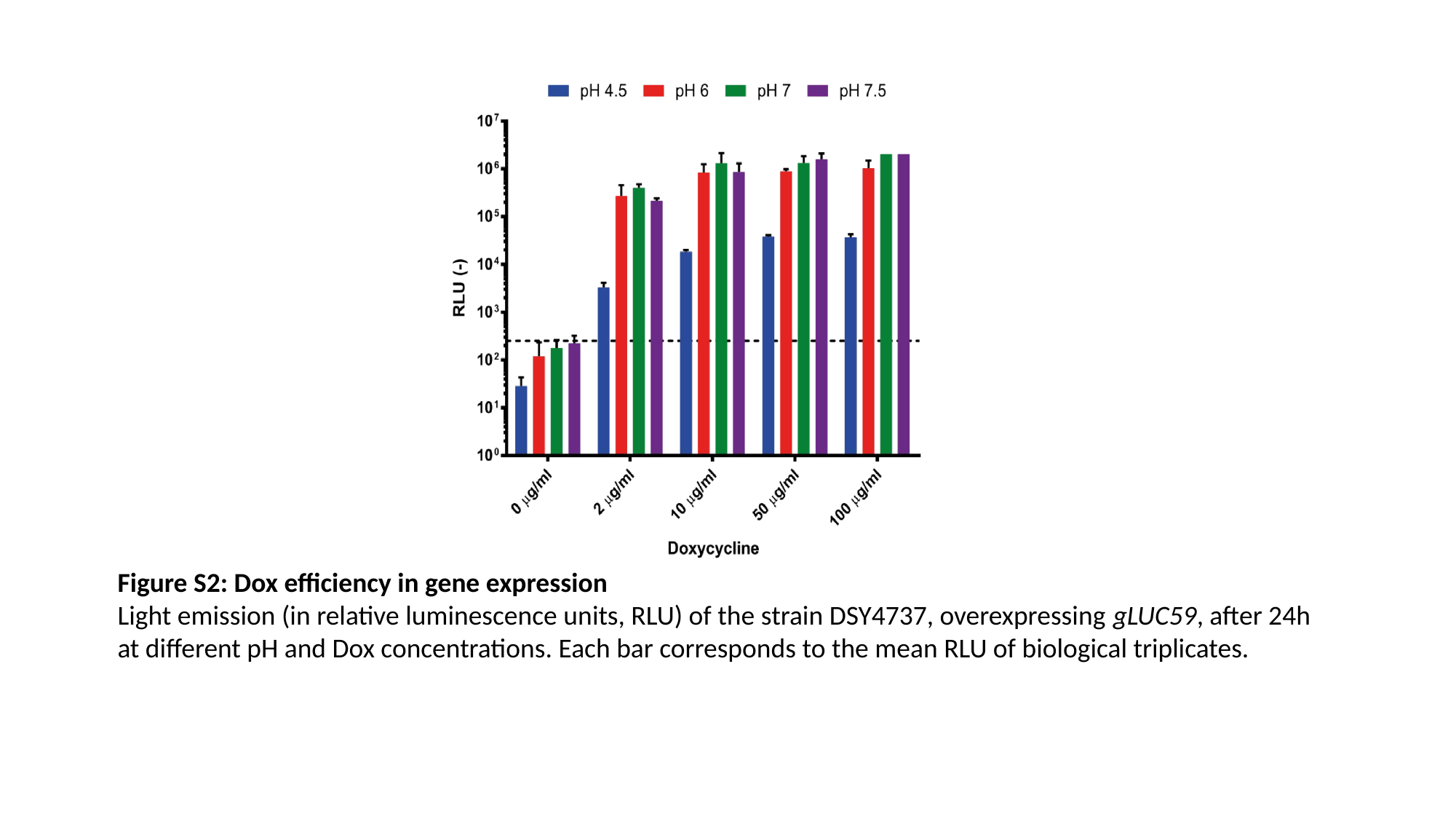

Figure S2: Dox efficiency in gene expression
Light emission (in relative luminescence units, RLU) of the strain DSY4737, overexpressing gLUC59, after 24h at different pH and Dox concentrations. Each bar corresponds to the mean RLU of biological triplicates.

## Slide 3
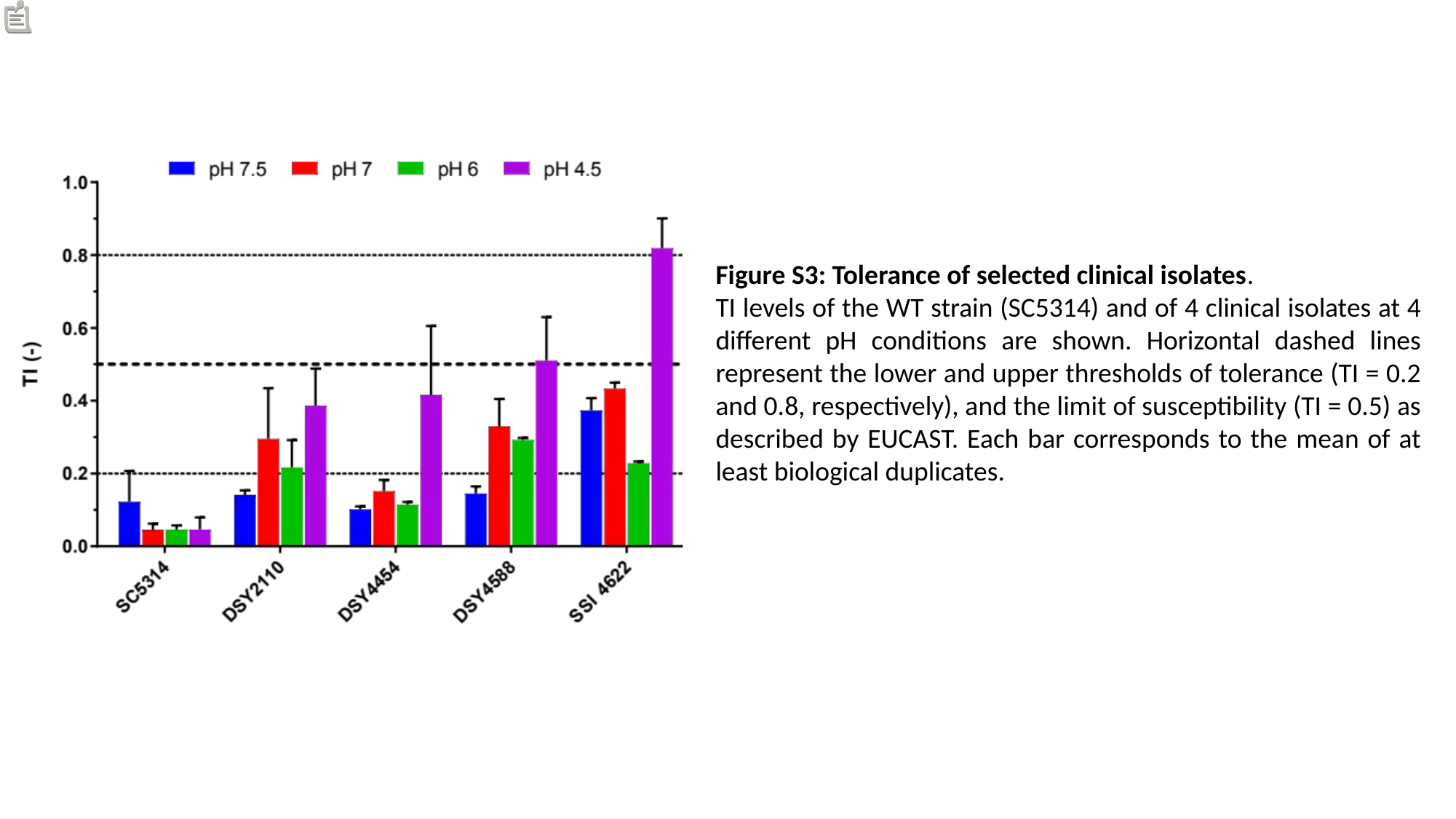

Figure S3: Tolerance of selected clinical isolates.
TI levels of the WT strain (SC5314) and of 4 clinical isolates at 4 different pH conditions are shown. Horizontal dashed lines represent the lower and upper thresholds of tolerance (TI = 0.2 and 0.8, respectively), and the limit of susceptibility (TI = 0.5) as described by EUCAST. Each bar corresponds to the mean of at least biological duplicates.

## Slide 4
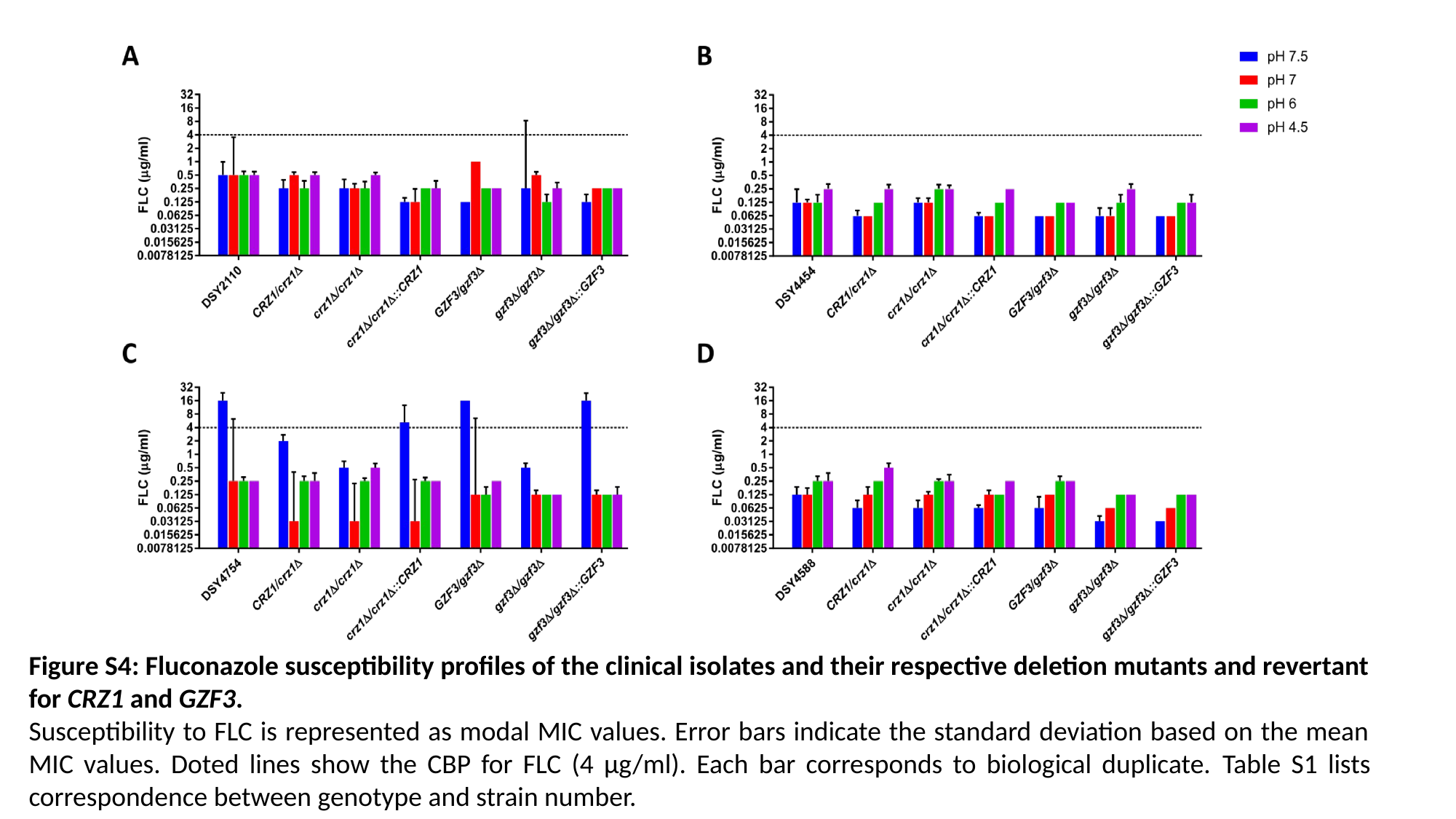

Figure S4: Fluconazole susceptibility profiles of the clinical isolates and their respective deletion mutants and revertant for CRZ1 and GZF3.
Susceptibility to FLC is represented as modal MIC values. Error bars indicate the standard deviation based on the mean MIC values. Doted lines show the CBP for FLC (4 µg/ml). Each bar corresponds to biological duplicate. Table S1 lists correspondence between genotype and strain number.

## Slide 5
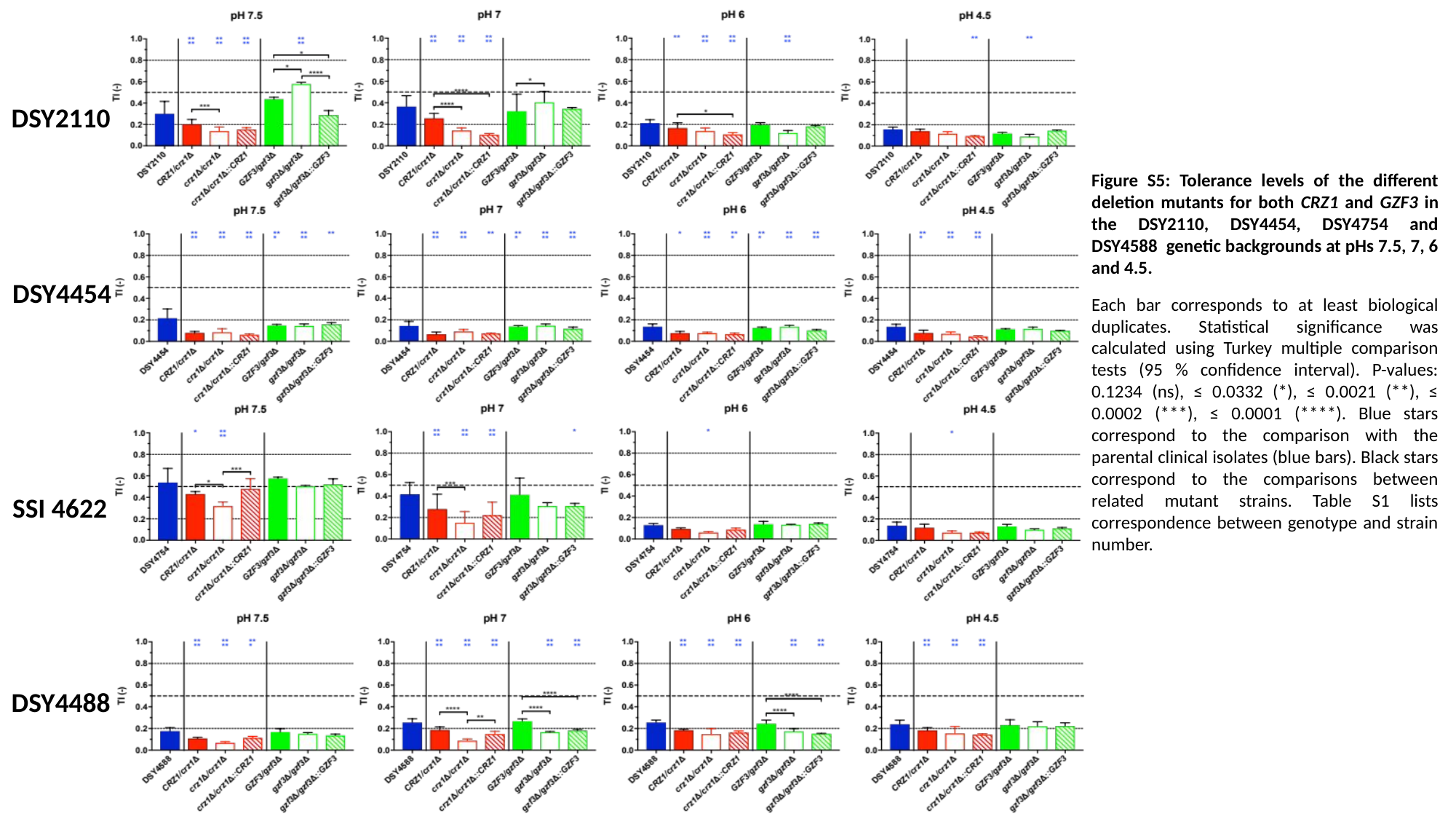

DSY2110
Figure S5: Tolerance levels of the different deletion mutants for both CRZ1 and GZF3 in the DSY2110, DSY4454, DSY4754 and DSY4588 genetic backgrounds at pHs 7.5, 7, 6 and 4.5.
Each bar corresponds to at least biological duplicates. Statistical significance was calculated using Turkey multiple comparison tests (95 % confidence interval). P-values: 0.1234 (ns), ≤ 0.0332 (*), ≤ 0.0021 (**), ≤ 0.0002 (***), ≤ 0.0001 (****). Blue stars correspond to the comparison with the parental clinical isolates (blue bars). Black stars correspond to the comparisons between related mutant strains. Table S1 lists correspondence between genotype and strain number.
DSY4454
SSI 4622
DSY4488

## Slide 6
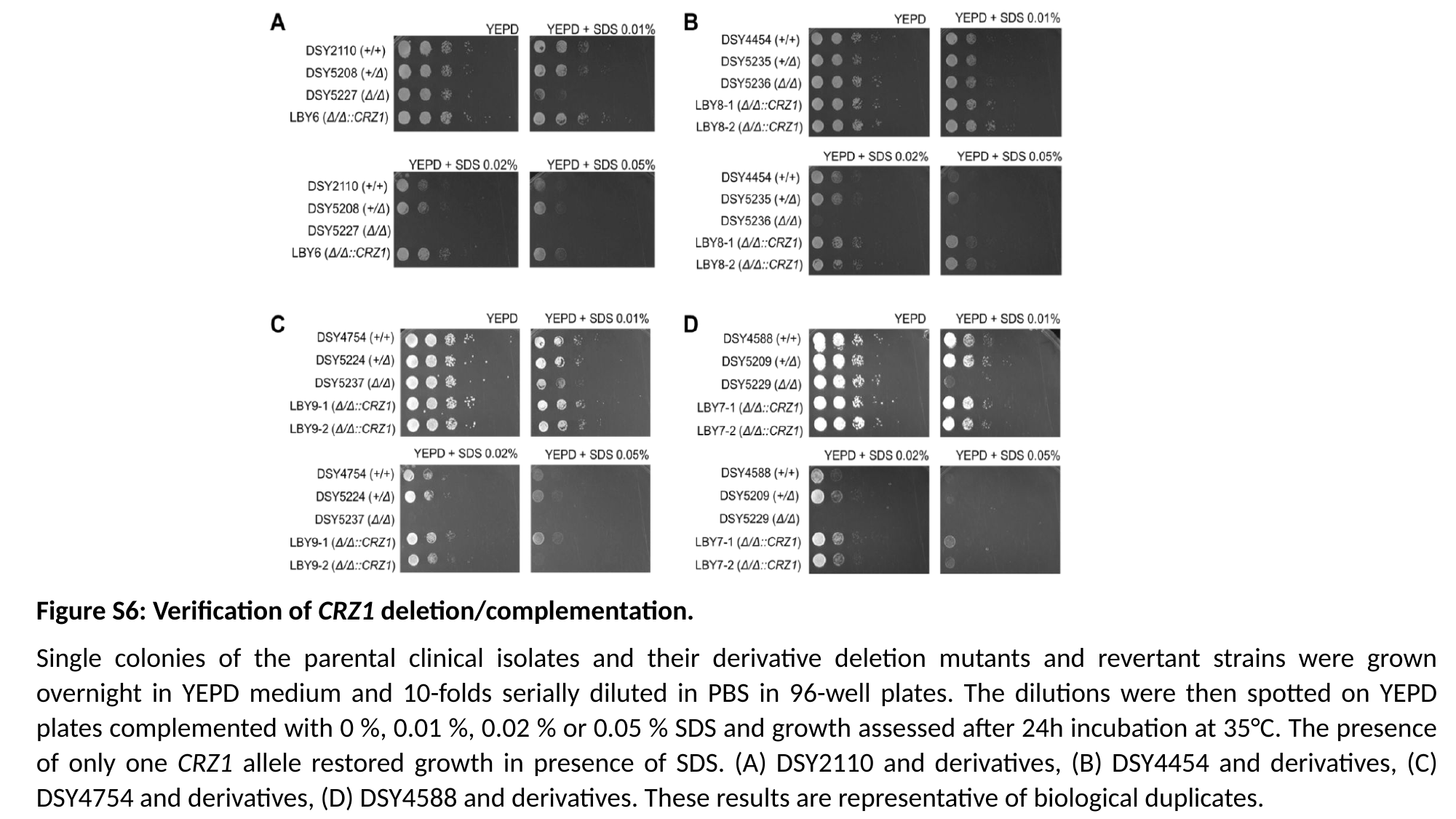

Figure S6: Verification of CRZ1 deletion/complementation.
Single colonies of the parental clinical isolates and their derivative deletion mutants and revertant strains were grown overnight in YEPD medium and 10-folds serially diluted in PBS in 96-well plates. The dilutions were then spotted on YEPD plates complemented with 0 %, 0.01 %, 0.02 % or 0.05 % SDS and growth assessed after 24h incubation at 35°C. The presence of only one CRZ1 allele restored growth in presence of SDS. (A) DSY2110 and derivatives, (B) DSY4454 and derivatives, (C) DSY4754 and derivatives, (D) DSY4588 and derivatives. These results are representative of biological duplicates.

## Slide 7
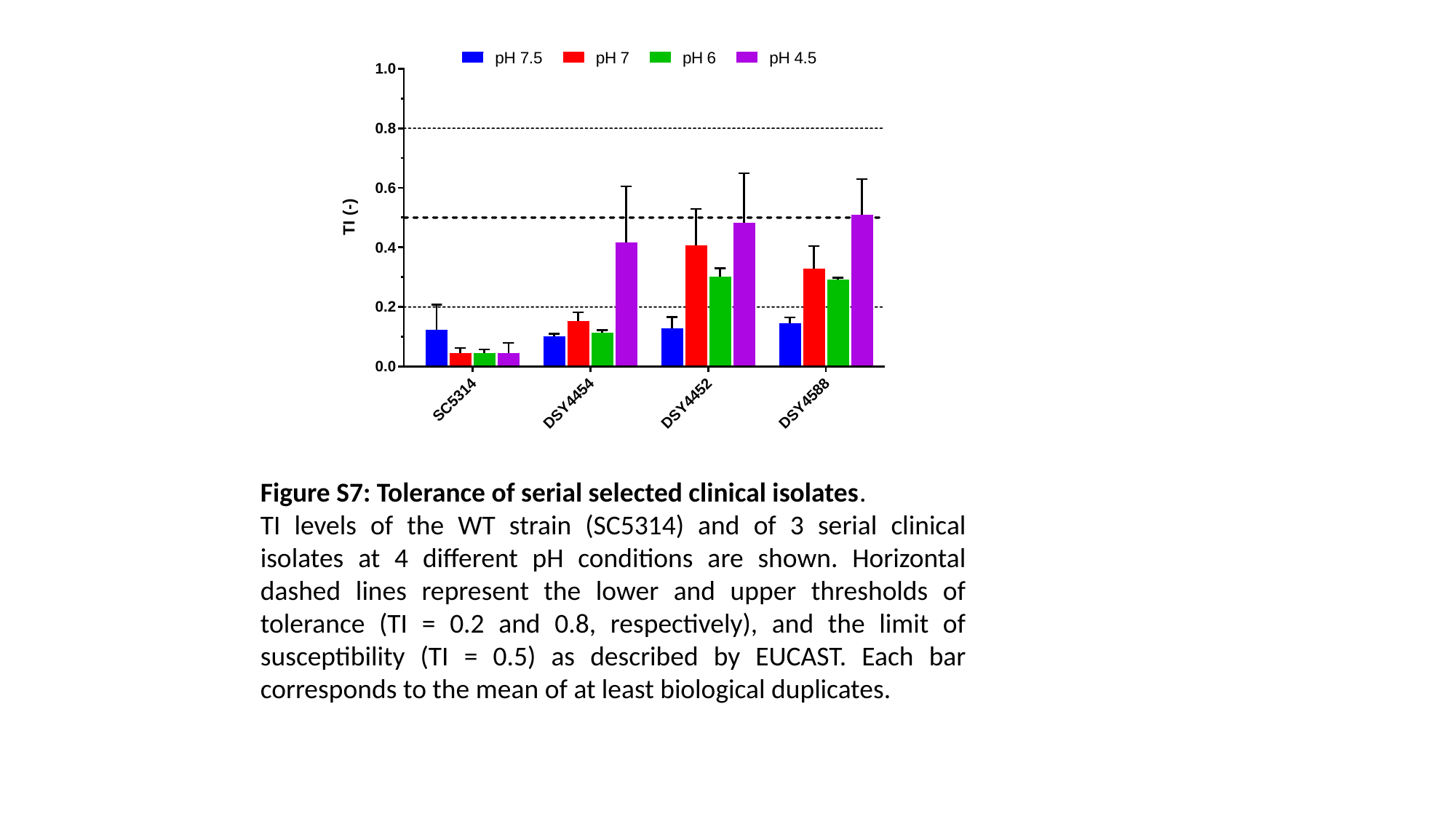

Figure S7: Tolerance of serial selected clinical isolates.
TI levels of the WT strain (SC5314) and of 3 serial clinical isolates at 4 different pH conditions are shown. Horizontal dashed lines represent the lower and upper thresholds of tolerance (TI = 0.2 and 0.8, respectively), and the limit of susceptibility (TI = 0.5) as described by EUCAST. Each bar corresponds to the mean of at least biological duplicates.

## Slide 8
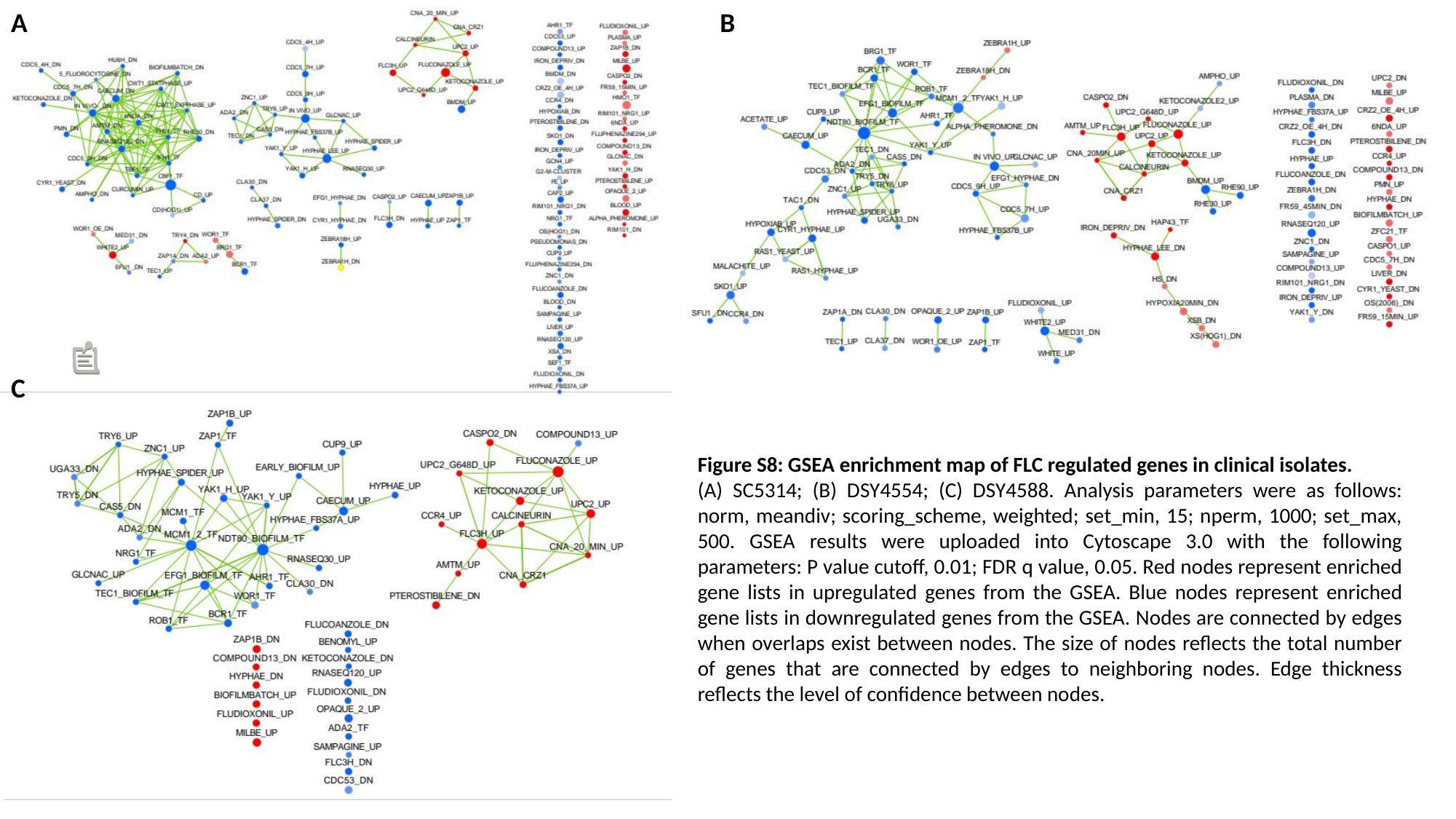

A
B
C
Figure S8: GSEA enrichment map of FLC regulated genes in clinical isolates.
(A) SC5314; (B) DSY4554; (C) DSY4588. Analysis parameters were as follows: norm, meandiv; scoring_scheme, weighted; set_min, 15; nperm, 1000; set_max, 500. GSEA results were uploaded into Cytoscape 3.0 with the following parameters: P value cutoff, 0.01; FDR q value, 0.05. Red nodes represent enriched gene lists in upregulated genes from the GSEA. Blue nodes represent enriched gene lists in downregulated genes from the GSEA. Nodes are connected by edges when overlaps exist between nodes. The size of nodes reflects the total number of genes that are connected by edges to neighboring nodes. Edge thickness reflects the level of confidence between nodes.

## Slide 9
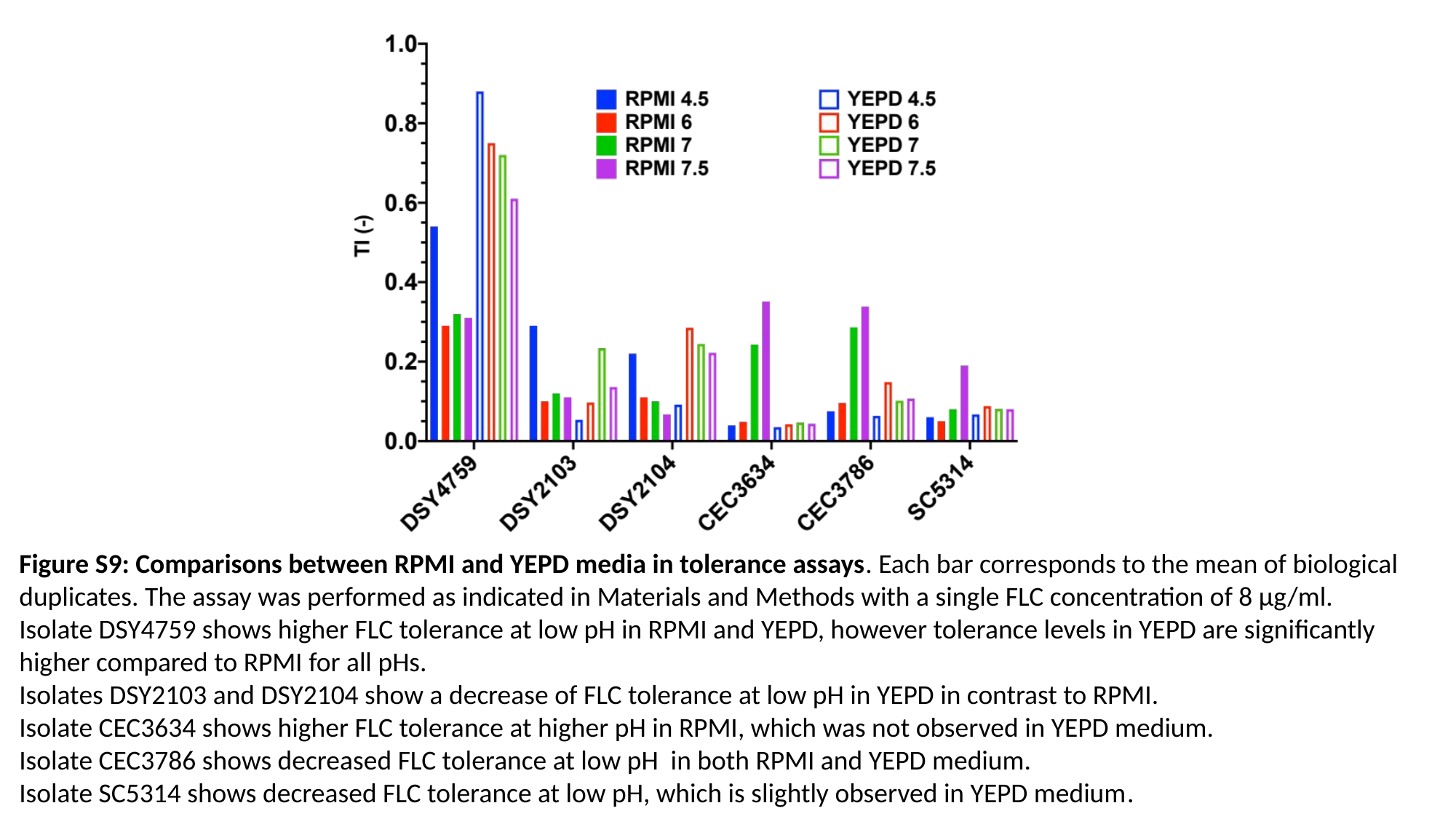

Figure S9: Comparisons between RPMI and YEPD media in tolerance assays. Each bar corresponds to the mean of biological duplicates. The assay was performed as indicated in Materials and Methods with a single FLC concentration of 8 µg/ml.
Isolate DSY4759 shows higher FLC tolerance at low pH in RPMI and YEPD, however tolerance levels in YEPD are significantly higher compared to RPMI for all pHs.
Isolates DSY2103 and DSY2104 show a decrease of FLC tolerance at low pH in YEPD in contrast to RPMI.
Isolate CEC3634 shows higher FLC tolerance at higher pH in RPMI, which was not observed in YEPD medium.
Isolate CEC3786 shows decreased FLC tolerance at low pH in both RPMI and YEPD medium.
Isolate SC5314 shows decreased FLC tolerance at low pH, which is slightly observed in YEPD medium.
