# Supplementary material for: Identification and Characterization of Mediators of Fluconazole Tolerance in Candida albicans
Source: Front Microbiol. 2020 Nov 11;11:591140. doi: 10.3389/fmicb.2020.591140 (PMC7686038; doi:10.3389/fmicb.2020.591140)
Supplement: Supplementary file 11 [file Table_1.DOCX]

**Table S1: Strains used in this study**

| **Lab ID** | **Alternative ID** | **Parental strain** | **Transformed plasmid** | **Genotype/phenotype** | **Reference** |
| --- | --- | --- | --- | --- | --- |
| DSY386 | SC5314 | NA^1^ |  | Reference strain | (1) |
| DSY2904 | BWP17 | NA |  | *ura3*Δ::λimm434/*ura3*Δ::λimm434,*iro1*∆::λimm434/*iro1*∆::λimm434, *his1*∆::*hisG*/*his1*∆::*hisG,* *arg4*∆::*hisG*/*arg4*∆::*hisG* | (2) |
| DSY3291 | SN152 | NA |  | *URA3*/*ura3*Δ::λimm434, *IRO1*/*iro1*Δ::λimm434, *his1*Δ::*hisG*/*his1*Δ::*hisG*, *arg4*Δ::*dpl200*/*arg4*Δ::*dpl200*, *leu2*Δ::*dpl200*/*leu2*Δ::*dpl200* | (3) |
| DSY2110 | 34-028-117 | Blood |  | Low-High phenotype^2^ | (4) |
| DSY4454 | 0526-6020 | Blood |  | Isolated from endocarditis patient (2005)^3^ | This study |
| DSY4452 | 1101-334 | Blood |  | Isolated from endocarditis patient (2011)^3^ | This study |
| DSY4588 | 13 0420 0673 | Blood |  | Isolated from endocarditis patient (2013)^3^ | This study |
| DSY4754 | SSI 4622 | NA |  | Weak Trailing^4^ | (5) |
| NA | CEC161 | BWP17 |  | *ura3*Δ::λimm434/*ura3*Δ::λimm434, *iro1*∆::λimm434/*iro1*∆::λimm434, *his1*Δ::*hisG*/*HIS1*, *arg4*Δ::*hisG*/*ARG4* | (6) |
| DSY4993 | CEC2907 | CEC161 | pNIMX | *ura3*Δ::λimm434/*ura3*Δ::λimm434, *iro1*∆::λimm434/*iro1*∆::λimm434, *his1*Δ::*hisG*/*HIS1*, *arg4*Δ::*hisG*/*ARG4*, *ADH1*/*adh1*::P*_TDH3_-cartTA*::*SAT1* | (7) |
| DSY4737 | CEC3083 | CEC2907 | CIp10-P_TET_-*gLUC59* | *ura3*Δ::λimm434/*ura3*Δ::λimm434, *iro1*∆::λimm434/*iro1*∆::λimm434, *his1*Δ::*hisG*/*HIS1*, *arg4*Δ::*hisG*/*ARG4*, *ADH1*/*adh1*::P*_TDH3_-cartTA*::*SAT1*, *RPS1*/*RPS1*::CIp10-P_TET_-*gLUC59* | (7) |
| NA | CPY2 | CEC2907 | pDS1945 | *ura3*Δ::λimm434/*ura3*Δ::λimm434, *iro1*∆::λimm434/*iro1*∆::λimm434, *his1*Δ::*hisG*/*HIS1*, *arg4*Δ::*hisG*/*ARG4*, *ADH1*/*adh1*::P*_TDH3_-cartTA*::*SAT1*, *RPS1*/*RPS1*::CIp10-P_TET_-GTW | This study |
| DSY4993 | CEC2907 | CEC161 | pNIMX | *ura3*Δ::λimm434/*ura3*Δ::λimm434, *iro1*∆::λimm434/*iro1*∆::λimm434, *his1*Δ::*hisG*/*HIS1*, *arg4*Δ::*hisG*/*ARG4*, *ADH1*/*adh1*::P*_TDH3_-cartTA*::*SAT1* | (7) |
| DSY4737 | CEC3083 | CEC2907 | CIp10-P_TET_-*gLUC59* | *ura3*Δ::λimm434/*ura3*Δ::λimm434, *iro1*∆::λimm434/*iro1*∆::λimm434, *his1*Δ::*hisG*/*HIS1*, *arg4*Δ::*hisG*/*ARG4*, *ADH1*/*adh1*::P*_TDH3_-cartTA*::*SAT1*, *RPS1*/*RPS1*::CIp10-P_TET_-*gLUC59* | (7) |
| NA | CPY21 | DSY4588 | pSFSUI | *ura3*Δ::FRT/*ura3*Δ::FRT | This study |
| NA | CPY23 | DSY4454 | pSFSUI | *ura3*Δ::FRT/*ura3*Δ::FRT | This study |
| NA | CPY25 | SC5314 | pSFSUI | *ura3*Δ::FRT/*ura3*Δ::FRT | This study |
| NA | CPY27 | DSY4754 | pSFSUI | *ura3*Δ::FRT/*ura3*Δ::FRT | This study |
| NA | CPY29 | DSY2110 | pSFSUI | *ura3*Δ::FRT/*ura3*Δ::FRT | This study |
| NA | CPY42^5^ | CPY29 | pNIMX | *ura3*Δ::FRT/*ura3*Δ::FRT, *ADH1*/*adh1*::P*_TDH3_*-*cartTA*::*SAT1* | This study |
| NA | CPY43^5^ | CPY23 | pNIMX | *ura3*Δ::FRT/*ura3*Δ::FRT, *ADH1*/*adh1*::P*_TDH3_*-*cartTA*::*SAT1* | This study |
| NA | CPY44^5^ | CPY21 | pNIMX | *ura3*Δ::FRT/*ura3*Δ::FRT, *ADH1*/*adh1*::P*_TDH3_*-*cartTA*::*SAT1* | This study |
| NA | CPY45^5^ | CPY27 | pNIMX | *ura3*Δ::FRT/*ura3*Δ::FRT, *ADH1*/*adh1*::P*_TDH3_*-*cartTA*::*SAT1* | This study |
| NA | "Wild type"^6^ | SN152 | NA | *URA3*/*ura3*∆, *IRO1*/*iro1*∆, *his1*Δ/*his1*Δ::*HIS1*, *arg4*Δ/Δ, *leu2*Δ/*leu2*Δ::*LEU2* | (8) |
| NA | TF068 | SN152 | NA | *URA3*/*ura3*∆, *IRO1*/*iro1*∆, *his1*∆/∆, *arg4*∆/∆, leu2∆/∆, *crz1*∆::*CdLEU2*/*crz1*∆::*CmHIS1*^7^ | (8) |
| NA | TF101 | SN152 | NA | *URA3*/*ura3*∆, *IRO1*/*iro1*∆, *his1*∆/∆, *arg4*∆/∆, leu2∆/∆, *gzf3*∆::*CdLEU2*/*gzf3*∆::*CmHIS1* | (8) |
| DSY5253 | EDY16-3 | TF068 | pSFSU1 | *ura3*∆/∆, *IRO1*/*iro1*∆, *his1*Δ/Δ, *arg4*Δ/Δ, *leu2*Δ/Δ, *crz1*∆::*CdLEU2*/*crz1*∆::*CmHIS1* | This study |
| DSY5254 | EDY17-3 | TF101 | pSFSU1 | *ura3*∆/∆, *IRO1*/*iro1*∆, *his1*Δ/Δ, *arg4*Δ/Δ, *leu2*Δ/Δ, *gzf3*∆::*CdLEU2*/*gzf3*∆::*CmHIS1* | This study |
| DSY5270 | EDY35 | "Wild type"^3^ | pSFSU1 | *ura3*∆/∆, *IRO1*/*iro1*∆, *his1*Δ/*his1*Δ::*HIS1*, *arg4*Δ/Δ, *leu2*Δ/*leu2*Δ::*LEU2* | This study |
| DSY5255 | EDY20-3 | EDY16-3 | pNIMX | *ura3*∆/∆, *IRO1*/*iro1*∆, *his1*Δ/Δ, *arg4*Δ/Δ, *leu2*Δ/Δ, *crz1*∆::*CdLEU2*/*crz1*∆::*CmHIS1*, *ADH1*/*adh1*Δ::P*_TDH3_*-*cartTA*::*SAT1* | This study |
| DSY5256 | EDY21-1 | EDY17-3 | pNIMX | *ura3*∆/∆, *IRO1*/*iro1*∆, *his1*Δ/Δ, *arg4*Δ/Δ, *leu2*Δ/Δ, *gzf3*∆::*CdLEU2*/*gzf3*∆::*CmHIS1*, *ADH1*/*adh1*Δ::P*_TDH3_*-*cartTA*::*SAT1* | This study |
| DSY5273 | EDY38 | EDY35 | pNIMX | *ura3*∆/∆, *IRO1*/*iro1*∆, *his1*Δ/*his1*Δ::*HIS1*, *arg4*Δ/Δ, *leu2*Δ/*leu2*Δ::*LEU2, ADH1*/*adh1*Δ::P*_TDH3_-cartTA*::*SAT1* | This study |
| DSY5274 | EDY39 | EDY38 | pDS1945 | *ura3*∆/∆, *IRO1*/*iro1*∆, *his1*Δ/*his1*Δ::*HIS1*, *arg4*Δ/Δ, *leu2*Δ/*leu2*Δ::*LEU2, ADH1*/*adh1*Δ::P*_TDH3_-cartTA*::*SAT1*, *RPS1*/*RPS1*::CIp10-P_TET_-GTW | This study |
| DSY5275 | EDY40 | EDY38 | pDS1997 | *ura3*∆/∆, *IRO1*/*iro1*∆, *his1*Δ/*his1*Δ::*HIS1*, *arg4*Δ/Δ, *leu2*Δ/*leu2*Δ::*LEU2, ADH1*/*adh1*Δ::P*_TDH3_-cartTA*::*SAT1*, *RPS1*/*RPS1*::CIp10-P_TET_-*GZF3* | This study |
| DSY5276 | EDY41 | EDY38 | pDS1998 | *ura3*∆/∆, *IRO1*/*iro1*∆, *his1*Δ/*his1*Δ::*HIS1*, *arg4*Δ/Δ, *leu2*Δ/*leu2*Δ::*LEU2, ADH1*/*adh1*Δ::P*_TDH3_-cartTA*::*SAT1*, *RPS1*/*RPS1*::CIp10-P_TET_-*CRZ1* | This study |
| DSY5271 | EDY36 | EDY20-3 | pDS1945 | *ura3*∆/∆, *IRO1*/*iro1*∆, *his1*Δ/Δ, *arg4*Δ/Δ, *leu2*Δ/Δ, *crz1*∆::*CdLEU2*/*crz1*∆::*CmHIS1*, *ADH1*/*adh1*Δ::P*_TDH3_*-*cartTA*::*SAT1*, *RPS1*/*RPS1*::CIp10-P_TET_-GTW | This study |
| DSY5257 | EDY22 | EDY20-3 | pDS1997 | *ura3*∆/∆, *IRO1*/*iro1*∆, *his1*Δ/Δ, *arg4*Δ/Δ, *leu2*Δ/Δ, *crz1*∆::*CdLEU2*/*crz1*∆::*CmHIS1*, *ADH1*/*adh1*Δ::P*_TDH3_*-*cartTA*::*SAT1*, *RPS1*/*RPS1*::CIp10-P_TET_-*GZF3* | This study |
| DSY5259 | EDY24 | EDY20-3 | pDS1998 | *ura3*∆/∆*, IRO1*/*iro1*∆, *his1*Δ/Δ, *arg4*Δ/Δ, *leu2*Δ/Δ, *crz1*∆::*CdLEU2*/*crz1*∆::*CmHIS1*, *ADH1*/*adh1*Δ::P*_TDH3_*-*cartTA*::*SAT1*, *RPS1*/*RPS1*::CIp10-P_TET_-*CRZ1* | This study |
| DSY5272 | EDY37 | EDY21-1 | pDS1945 | *ura3*∆/∆, *IRO1*/*iro1*∆, *his1*Δ/Δ, *arg4*Δ/Δ, *leu2*Δ/Δ, *gzf3*∆::*CdLEU2*/*gzf3*∆::*CmHIS1*, *ADH1*/*adh1*Δ::P*_TDH3_*-*cartTA*::*SAT1*, *RPS1*/*RPS1*::CIp10-P_TET_-GTW | This study |
| DSY5260 | EDY25 | EDY21-1 | pDS1997 | *ura3*∆/∆, *IRO1*/*iro1*∆, *his1*Δ/Δ, *arg4*Δ/Δ, *leu2*Δ/Δ, *gzf3*∆::*CdLEU2*/*gzf3*∆::*CmHIS1*, *ADH1*/*adh1*Δ::P*_TDH3_*-*cartTA*::*SAT1*, *RPS1*/*RPS1*::CIp10-P_TET_-*GZF3* | This study |
| DSY5258 | EDY23 | EDY21-1 | pDS1998 | *ura3*∆/∆, *IRO1*/*iro1*∆, *his1*Δ/Δ, *arg4*Δ/Δ, *leu2*Δ/Δ, *gzf3*∆::*CdLEU2*/*gzf3*∆::*CmHIS1*, *ADH1*/*adh1*Δ::P*_TDH3_*-*cartTA*::*SAT1*, *RPS1*/*RPS1*::CIp10-P_TET_-*CRZ1* | This study |
| DSY5263 | EDY28 | EDY16-3 | pED20-7 | *ura3*∆/∆, *IRO1*/*iro1*∆, *his1*Δ/Δ, *arg4*Δ/Δ, *leu2*Δ/Δ, *crz1*∆::*CdLEU2*/*crz1*∆::*CmHIS1*, *gzf3*∆/∆ | This study |
| DSY5265 | EDY30 | EDY28 | pNIMX | *ura3*∆*/*∆, *IRO1*/*iro1*∆, *his1*Δ/Δ, *arg4*Δ/Δ, *leu2*Δ/Δ, *crz1*∆::*CdLEU2*/*crz1*∆::*CmHIS1*, *gzf3*∆/∆, *ADH1*/*adh1*Δ::P*_TDH3_*-c*artTA*::*SAT1* | This study |
| DSY5269 | EDY34 | EDY30 | pDS1945 | *ura3*∆/∆, *IRO1*/*iro1*∆, *his1*Δ/Δ, *arg4*Δ/Δ, *leu2*Δ/Δ, *crz1*∆::*CdLEU2*/*crz1*∆::*CmHIS1*, *gzf3*∆/∆, *ADH1*/*adh1*Δ::P*_TDH3_*-*cartTA*::*SAT1*, *RPS1*/*RPS1*::CIp10-P_TET_-GTW | This study |
| DSY5267 | EDY32 | EDY30 | pDS1997 | *ura3*∆/∆, *IRO1*/*iro1*∆, *his1*Δ/Δ, *arg4*Δ/Δ, *leu2*Δ/Δ, *crz1*∆::*CdLEU2*/*crz1*∆::*CmHIS1*, *gzf3*∆/∆, *ADH1*/*adh1*Δ::P*_TDH3_*-*cartTA*::*SAT1*, *RPS1*/*RPS1*::CIp10-P_TET_-*GZF3* | This study |
| DSY5266 | EDY31 | EDY30 | pDS1998 | *ura3*∆/∆, *IRO1*/*iro1*∆, *his1*Δ/Δ, *arg4*Δ/Δ, *leu2*Δ/Δ, *crz1*∆::*CdLEU2*/*crz1*∆::*CmHIS1*, *gzf3*∆/∆, *ADH1*/*adh1*Δ::P*_TDH3_*-*cartTA*::*SAT1*, *RPS1*/*RPS1*::CIp10-P_TET_-*CRZ1* | This study |
| DSY5208 | NA | DSY2110 | pED19-2 | *CRZ1/*crz1∆::FRT | This study |
| DSY5227 | NA | DSY5208 | pED19-2 | *crz1*∆::FRT/*crz1*∆::FRT | This study |
| NA | LBY6^8^ | DSY5227 | pLB1 | *crz1*∆/*crz1*∆::FRT::*CRZ1* | This study |
| DSY5210 | NA | DSY2110 | pED20-7 | *GZF3*/*gzf3*∆::FRT | This study |
| DSY5230 | NA | DSY5210 | pED20-7 | *gzf3*∆::FRT/*gzf3*∆::FRT | This study |
| DSY5279^1^ | NA | DSY5230 | pET3 | *gzf3*∆::FRT/*gzf3*∆::*GZF3* | This study |
| DSY5235 | NA | DSY4454 | pED19-2 | *CRZ1/crz1*∆::FRT | This study |
| DSY5236 | NA | DSY5235 | pED19-2 | *crz1*∆::FRT/*crz1*∆::FRT | This study |
| NA | LBY8^8^ | DSY5236 | pLB1 | *crz1*∆/*crz1*∆::FRT::*CRZ1* | This study |
| DSY5225 | NA | DSY4457 | pED20-7 | *GZF3*/*gzf3*∆::FRT | This study |
| DSY5231 | NA | DSY5225 | pED20-7 | *gzf3*∆::FRT/*gzf3*∆::FRT | This study |
| DSY5280^1^ | NA | DSY5231 | pET3 | *gzf3*∆::FRT/*gzf3*∆::*GZF3* | This study |
| DSY5209 | NA | DSY4588 | pED19-2 | *CRZ1/crz1*∆::FRT | This study |
| DSY5229 | NA | DSY5209 | pED19-2 | *crz1*∆::FRT/*crz1*∆::FRT | This study |
| NA | LBY7^8^ | DSY5229 | pLB1 | *crz1*∆/*crz1*∆::FRT::*CRZ1* | This study |
| DSY5217 | NA | DSY4588 | pED20-7 | *GZF3*/*gzf3*∆::FRT | This study |
| DSY5252 | NA | DSY5217 | pET2-1 | *gzf3*∆::FRT/*gzf3*∆::FRT | This study |
| DSY5277^1^ | NA | DSY5252 | pET3 | *gzf3*∆::FRT/*gzf3*∆::*GZF3* | This study |
| DSY5224 | NA | DSY4754 | pED19-2 | *CRZ1/crz1*∆::FRT | This study |
| DSY5237 | NA | DSY5224 | pED19-2 | *crz1*∆::FRT/*crz1*∆::FRT | This study |
| NA | LBY9^8^ | DSY5237 | pLB1 | *crz1*∆/*crz1*∆::FRT::*CRZ1* | This study |
| DSY5218 | NA | DSY4754 | pED20-7 | *GZF3*/*gzf3*∆::FRT | This study |
| DSY5239 | NA | DSY5218 | pED20-7 | *gzf3*∆::FRT/*gzf3*∆::FRT | This study |
| DSY5278 | NA | DSY5239 | pET3 | *gzf3*∆::FRT/*gzf3*∆::*GZF3* | This study |

^1^Not available

^2^As described by Marr *et al.* (4)

^3^Isolates were isolated from the same patient

^4^Information provided by M.C. Arendrup

^5^The derivative strains transformed with the CIp10-P_TET_-GOI plasmids are detailed in Supplementary Table 1

^6^Refers to Homann’s “Wild type” strain (8)

^7^*Cd* = *C. dubliniensis*, *Cm* = *C. maltosa*

^8^Revertant were built by reinsertion of the SC5314 allele at the deleted locus

**References**

1. Fonzi W, Irwin M. Isogenic strain construction and gene mapping in Candida albicans. *Genetics* (1993) 134:717-28. doi: 10.1007/978-1-60327-151-6.

2. Wilson RB, Davis D, Mitchell AP. *Rapid hypothesis testing with Candida albicans through gene disruption with short homology regions.* Journal of bacteriology(1999). p. 1868-74.

3. Noble SM, Johnson AD. *Strains and Strategies for Large-Scale Gene Deletion Studies of the Diploid Human Fungal Pathogen Candida albicans* Eukaryotic Cell(2005). p. 298-309.

4. Marr KA, Rustad TR, Rex JH, White C. The Trailing End Point Phenotype in Antifungal Susceptibility Testing Is pH Dependent. *Antimicrobial agents and chemotherapy* (1999) 43:1383-6. doi: 10.1128/AAC.43.6.1383.

5. Astvad KMTT, Johansen HK, Røder BL, Rosenvinge FS, Knudsen JD, Lemming L, et al. *Update from a 12-Year Nationwide Fungemia Surveillance: Increasing Intrinsic and Acquired Resistance Causes Concern*. In: Diekema DJ, editor. Journal of Clinical Microbiology(2017). p. 1-15.

6. Firon A, Aubert S, Iraqui I, Guadagnini S, Goyard S, Prévost MCC, et al. *The SUN41 and SUN42 genes are essential for cell separation in Candida albicans*. Molecular Microbiology(2007). p. 1256-75.

7. Chauvel M, Nesseir A, Cabral V, Znaidi S, Goyard S, Bachellier-Bassi S, et al. A Versatile Overexpression Strategy in the Pathogenic Yeast Candida albicans: Identification of Regulators of Morphogenesis and Fitness. *PLoS ONE* (2012). doi: 10.1371/journal.pone.0045912.

8. Homann OR, Dea J, Noble SM, Johnson AD. A phenotypic profile of the Candida albicans regulatory network. *PLoS Genetics* (2009) 5. doi: 10.1371/journal.pgen.1000783.
